# Supplementary material for: MOSHPIT: Accessible, reproducible metagenome data science on the QIIME 2 framework
Source: Cell Genom. 2026 Sep 24;6(10):101366. doi: 10.1016/j.xgen.2026.101366 (PMC13689502; doi:10.1016/j.xgen.2026.101366)
Supplement: Supplementary file 1 [file mmc1.pdf]

Cell Genomics, Volume *6*

Supplemental information

MOSHPIT: Accessible, reproducible metagenome data science on the QIIME 2 framework

Michal Ziemski, Liz Gehret, Anthony Simard, Santiago Castro Dau, Vinzent Risch, Doriela Grabocka, Christos Matzoros, Colin Wood, Paula Momo Cabrera, Rodrigo Hernández-Velázquez, Milo R. Schärer, Felicia Sandberg, Chloe Herman, Keegan Evans, Michael S. Robeson II, Evan Bolyen, J. Gregory Caporaso, and Nicholas A. Bokulich

Supplemental Figures

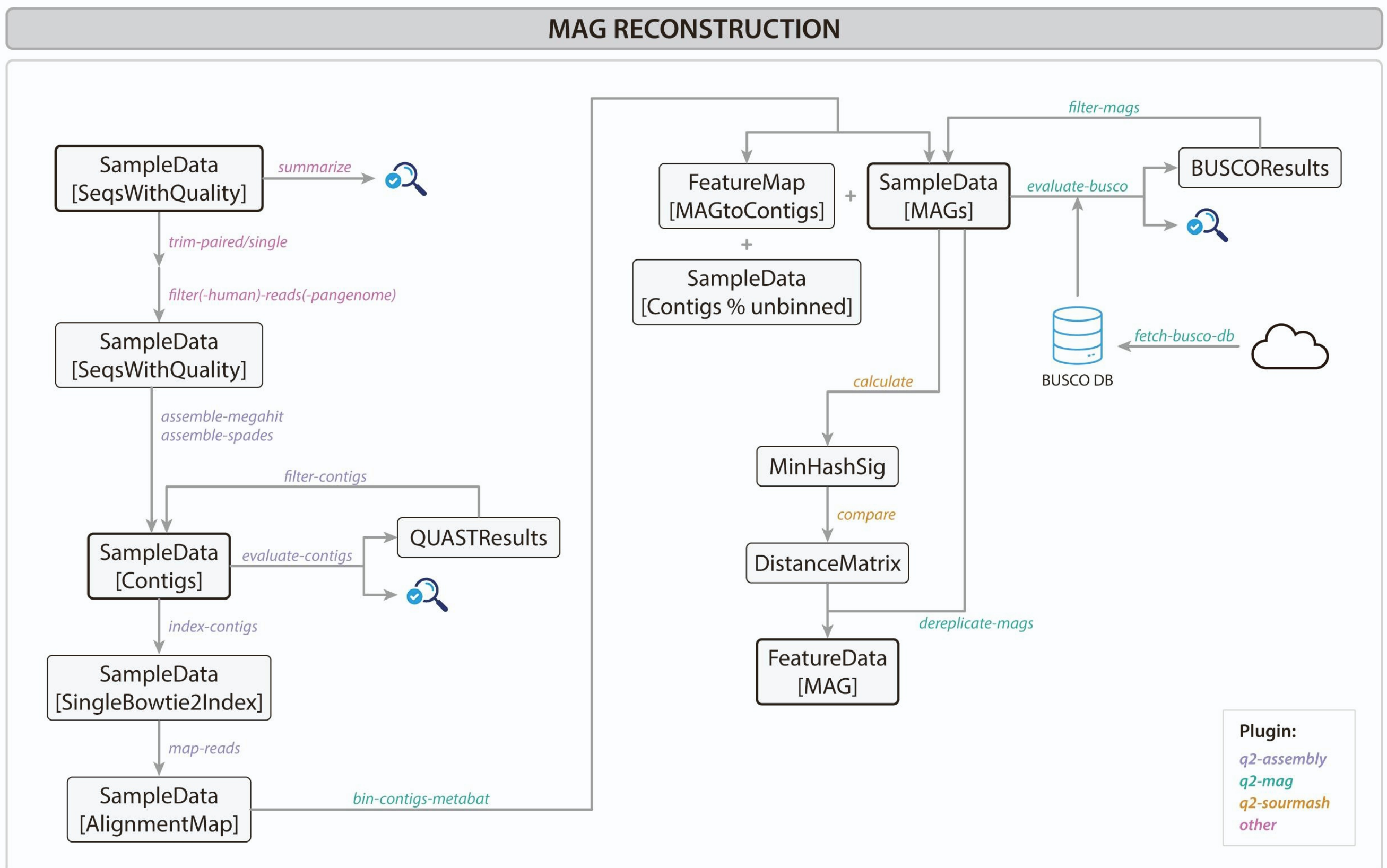

Figure S1: MOSHPIT MAG reconstruction workflow, related to Figure 1A. MAG reconstruction workflow comprises all the steps which are required to recover Metagenome-Assembled Genomes from reads, including their dereplication into a non-redundant set of genomes. Initially, reads can be quality controlled/filtered to remove potential contaminants (e.g. host reads). On top of the *filter-reads* action (available through the existing q2-quality-control plugin), we developed the new *filter-reads-human-pangenome* action (as part of the q2-quality-control plugin) which allows users to automatically construct an index comprised of the GRCh38 reference human genome and the most recently published human pangenome[1] and use it to filter out human host reads. Following the quality control steps, metagenome assembly can be performed using one of the two actions: *assemble-megahit* or *assemble-spades*, which will use the respective assembler (MEGAHIT[2] or SPAdes[3]) to reconstruct the contigs. Those can then be quality controlled using the *evaluate-quast* [4] or *evaluate-contigs* actions and indexed with the *index-contigs* action. In preparation for the binning step, the original reads can then be mapped to the contigs (*map-reads* action) using the index generated in the previous step. These contigs are then used as an input to the *bin-contigs-metabat* action, which will use MetaBat 2[5] to form the bins. Those can then be quality controlled with the *evaluate-busco* action[6]. Following binning, construction of a non-redundant set of genomes may be performed by first generating a distance matrix for the given genome set and using it to cluster genomes together, selecting one representative per cluster. Our workflow makes use of the per-MAG MinHash signatures generated through the *calculate* action (q2-sourmash plugin[7]), followed by the *compare* action (from the same plugin) to generate the distance matrix. The resulting table can then be used as input to our *dereplicate-mags* action which will find MAG clusters based on a distance threshold using hierarchical clustering with Ward linkage. It will then select the longest/most complete genome in each cluster (depending on whether completeness information was provided) to be its representative and return those non-redundant genomes together with a presence-absence table indicating which samples they were found in. Finally, to estimate the abundance of those genomes in the original samples, the dereplicated MAGs

can be indexed using the *index-derep-mags* action (q2-assembly), followed by read mapping using the *map-reads* action. The resulting maps, together with genome lengths (obtained via the *get-feature-lengths* action), can be input into the *estimate-abundance* action. This action will count the reads mapped to every MAG (while applying filtering parameters like minimal mapping quality, read length or base quality) and convert the counts using one of the available metrics like RPKM or TPM. Those metrics will then be returned as a feature table which can be used with any of the existing downstream QIIME 2 actions.

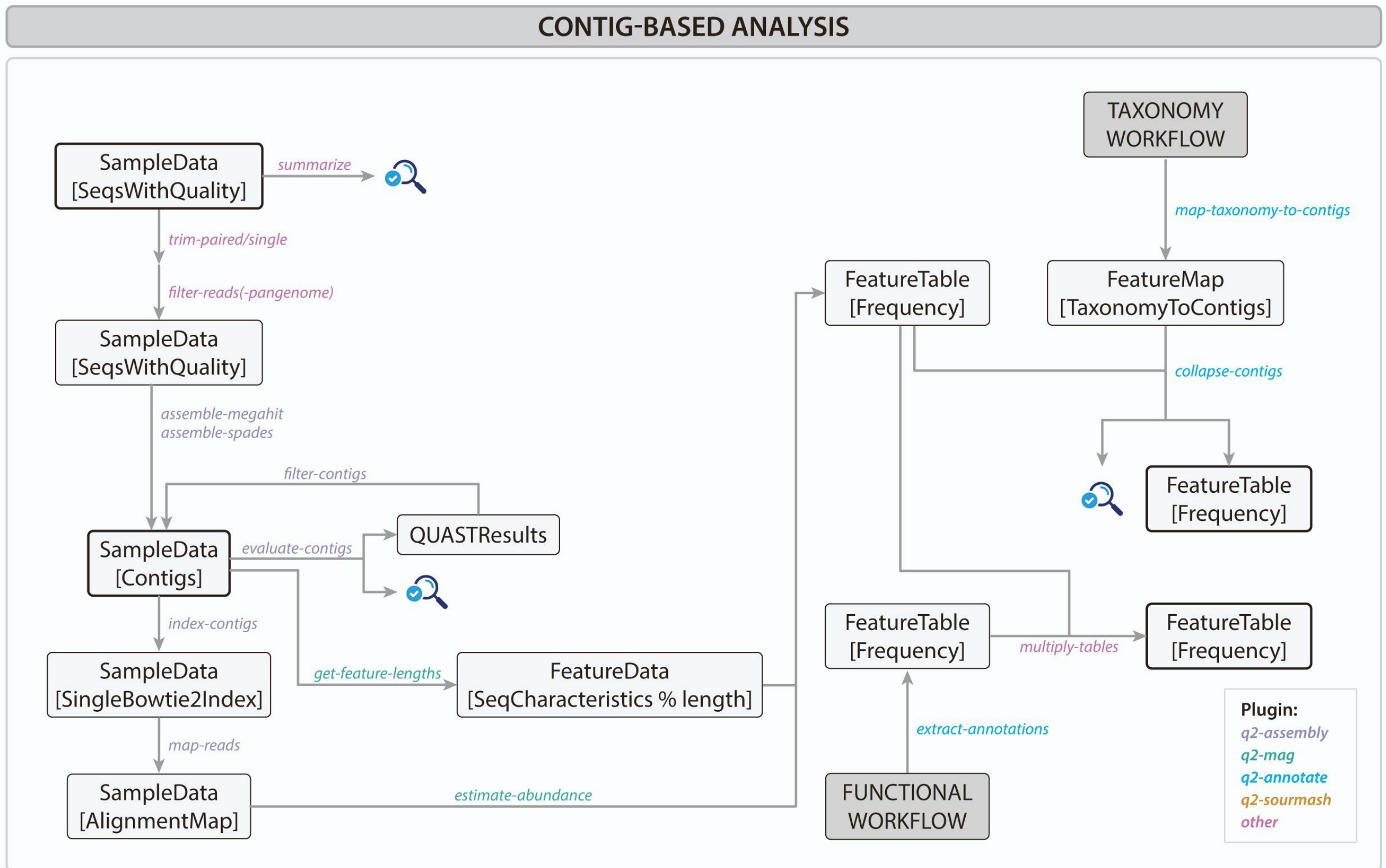

Figure S2: MOSHPIT contig-based analysis workflow, related to Figure 1A. Contig-based analysis workflow provides insight into community composition and functional profile already at the stage of genome assembly, without the need for contig binning and MAG reconstruction. The *estimate-abundance* action provides abundance estimates for each contig in a given sample and, when followed by the *collapse-contigs* action, can provide an early glimpse into the taxonomic composition of each sample. Additionally, functional profiles can be obtained by annotating contigs using one of the *search-orthologs-diamond* or *search-orthologs-hmmer* actions, followed by *map-eggnog*, *extract-annotations* and *multiply-tables*.

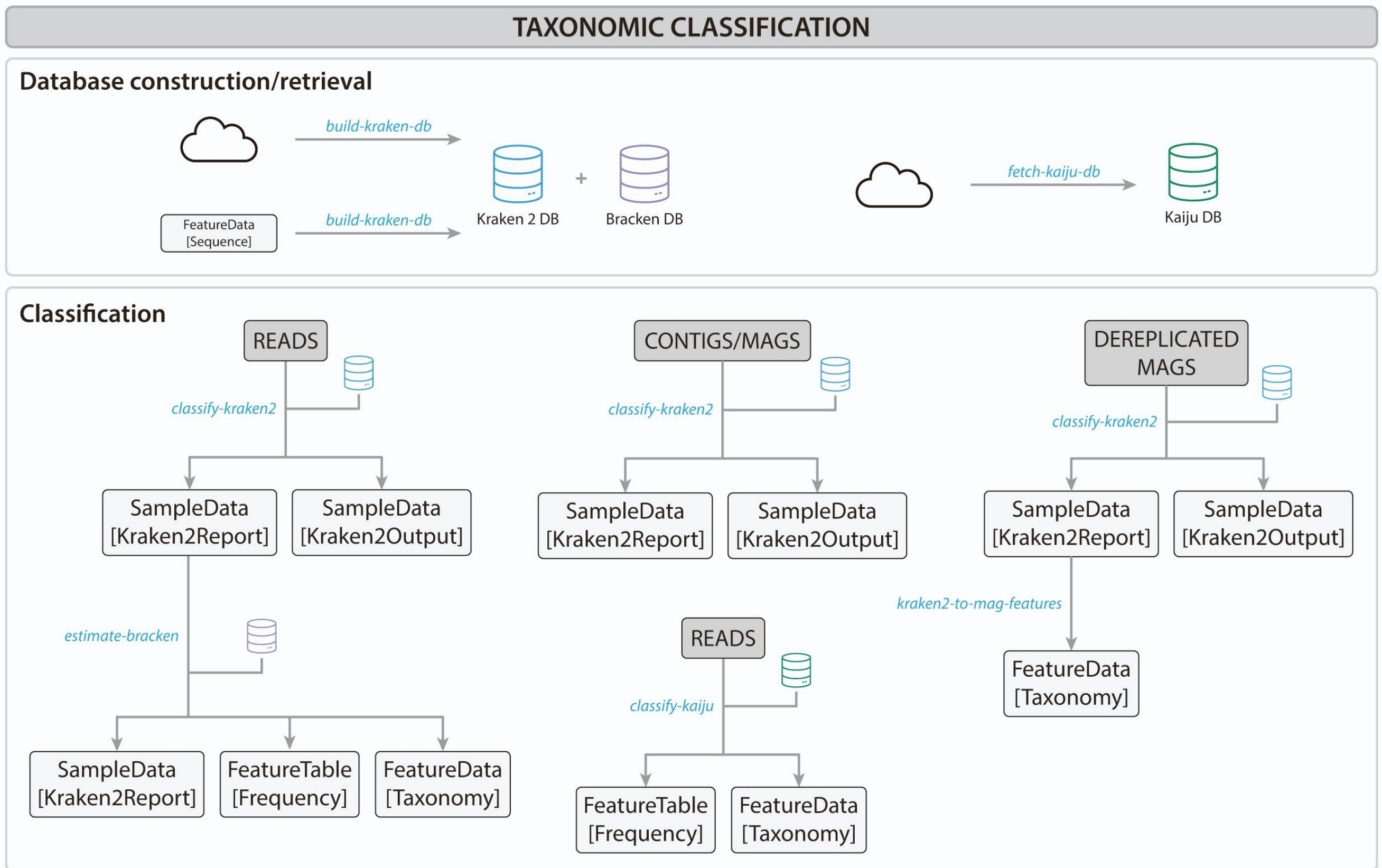

Figure S3: MOSHPIT taxonomic classification workflow, related to Figure 1A. Within the MOSHPIT plugin suite we provide two commonly used taxonomic classifiers: Kraken 2[8] together with its Bracken companion[9] used for read abundance correction and Kaiju for taxonomic classification[10]. We include actions which will allow users to easily fetch any of the pre-built databases for both of those classifiers (*build-kraken-db* and *fetch-kaiju-db* actions) and build custom Kraken 2 databases (*build-kraken-db*). We are planning on including an action to build custom Kaiju databases at a later point. Kraken 2 can be used to classify reads, contigs and MAGs (before and after dereplication) by using the *classify-kraken2* action. The reports obtained for the input reads can then be used as an input to the *estimate-bracken* action which will generate a feature table and a corresponding taxonomy. Furthermore, taxonomy features may be retrieved for the results generated from dereplicated MAGs by using the *kraken2-to-mag-features* - those can later be used to analyze the feature table obtained through MAG abundance estimation described in the previous section. The Kaiju classifier can be used to classify reads directly using the *classify-kaiju* action which will produce a feature table and a corresponding taxonomy, similar to the *classify-kraken2* action when reads are used as an input.

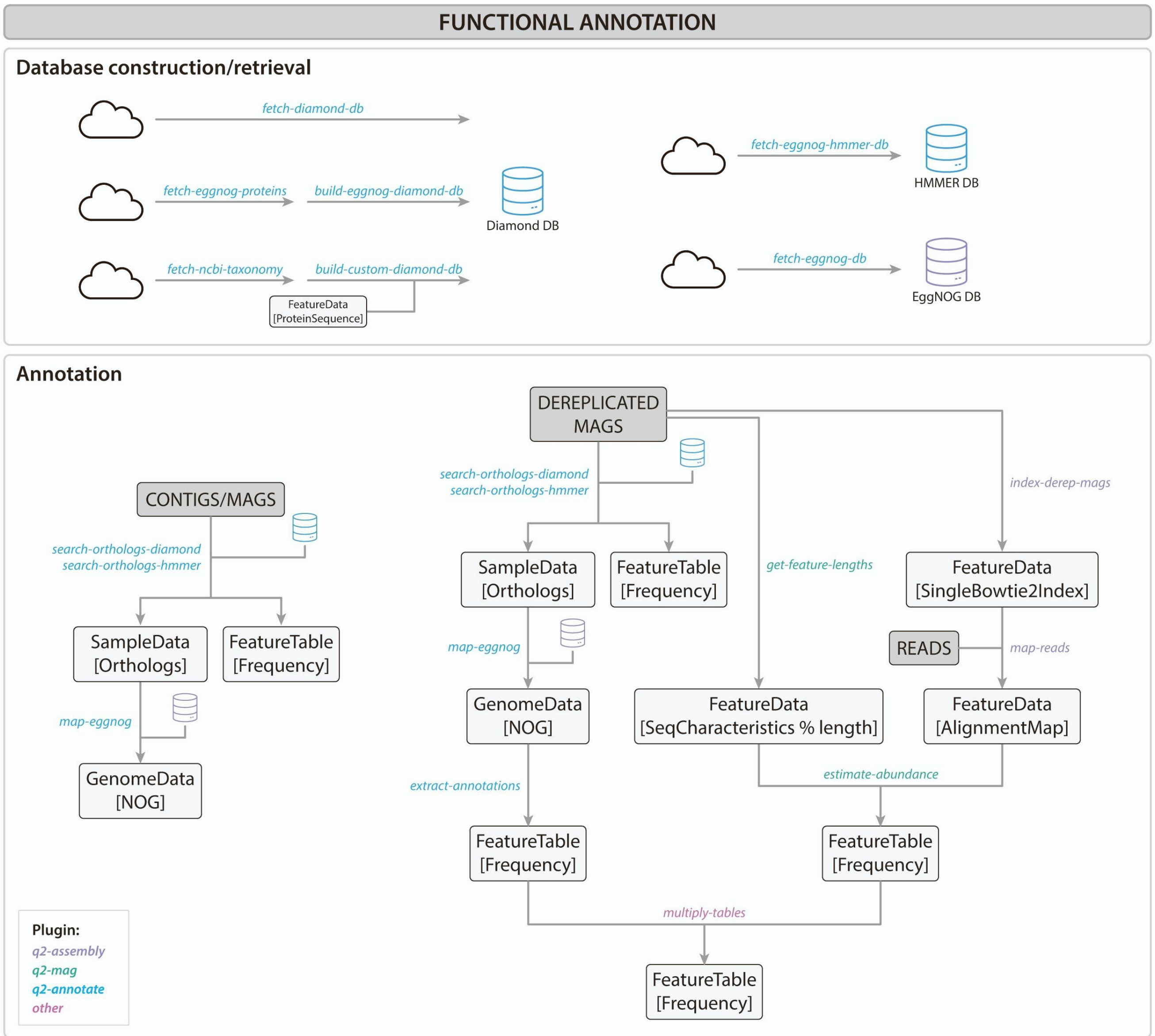

Figure S4. MOSHPIT functional annotation workflow, related to Figure 1A. The functional annotation workflow consists of various actions included in the q2-annotate plugin. The majority of these wrap EggNOG-mapper[11] to provide various options for functional annotation. We included several actions for retrieval/construction of databases for functional annotation, e.g., Diamond/HMMER databases for ortholog search and the EggNOG database used for ortholog annotation. Both contigs and MAGs (before and after dereplication) can be searched for protein orthologs using either *search-orthologs-diamond* or *search-orthologs-hmmer* actions. The resulting ortholog tables can then be annotated using the *map-eggnog* action and any specific annotation (e.g.: CAZymes or KEGG pathways) can be expanded into an independent feature table through the use of the *extract-annotations* action. This table can be passed as input into any other action accepting regular feature tables, e.g. to perform diversity analyses. Furthermore, when annotating the dereplicated MAGs (represented by the FeatureData[MAG] semantic type) this table can be combined with the feature table obtained from the MAG abundance estimation step using the *multiply-tables* action. This will generate a feature table with annotation counts normalized by the MAG abundances in each sample.

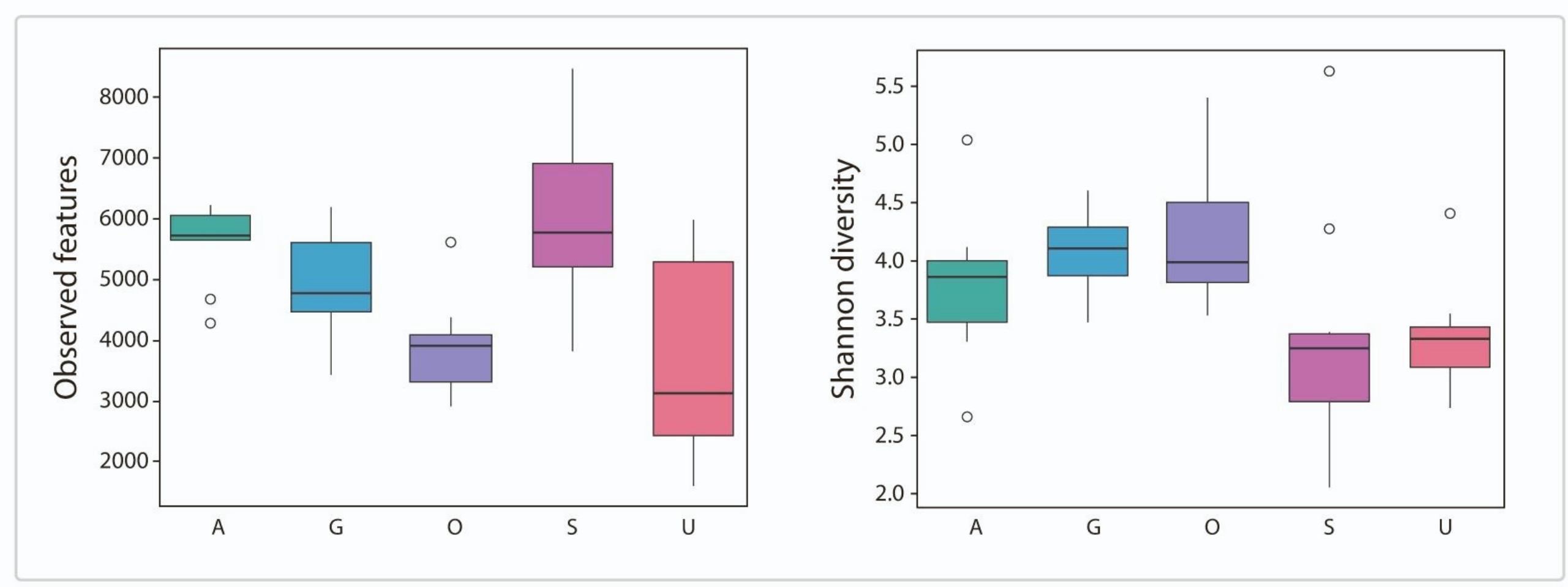

Figure S5: **Alpha diversity analysis of each body site (A: airways, G: gastrointestinal tract, O: oral cavity, S: skin, U: urogenital tract), related to Figure 2.**

Supplemental References

1. Liao, W.-W., Asri, M., Ebler, J., Doerr, D., Haukness, M., Hickey, G., Lu, S., Lucas, J.K., Monlong, J., Abel, H.J., et al. (2023). A draft human pangenome reference. Nature *617*, 312–324. https://doi.org/10.1038/s41586-023-05896-x.

2. Li, D., Liu, C.M., Luo, R., Sadakane, K., and Lam, T.W. (2015). MEGAHIT: An ultra-fast single-node solution for large and complex metagenomics assembly via succinct de Bruijn graph. Bioinformatics *31*, 1674–1676. https://doi.org/10.1093/bioinformatics/btv033.

3. Nurk, S., Meleshko, D., Korobeynikov, A., and Pevzner, P.A. (2017). MetaSPAdes: A new versatile metagenomic assembler. Genome Research *27*, 824–834. https://doi.org/10.1101/gr.213959.116.

4. Mikheenko, A., Saveliev, V., and Gurevich, A. (2016). MetaQUAST: Evaluation of metagenome assemblies. Bioinformatics *32*, 1088–1090. https://doi.org/10.1093/bioinformatics/btv697.

5. Kang, D.D., Li, F., Kirton, E., Thomas, A., Egan, R., An, H., and Wang, Z. (2019). MetaBAT 2: An adaptive binning algorithm for robust and efficient genome reconstruction from metagenome assemblies. PeerJ *2019*. https://doi.org/10.7717/peerj.7359.

6. Manni, M., Berkeley, M.R., Seppey, M., and Zdobnov, E.M. (2021). BUSCO: Assessing Genomic Data Quality and Beyond. Current Protocols *1*, e323. https://doi.org/10.1002/cpz1.323.

7. Pierce, N.T., Irber, L., Reiter, T., Brooks, P., and Brown, C.T. (2019). Large-scale sequence comparisons with *sourmash*. Preprint at F1000Research, https://doi.org/10.12688/f1000research.19675.1 https://doi.org/10.12688/f1000research.19675.1.

8. Wood, D.E., Lu, J., and Langmead, B. (2019). Improved metagenomic analysis with Kraken 2. Genome Biology *20*, 257. https://doi.org/10.1186/s13059-019-1891-0.

9. Lu, J., Breitwieser, F.P., Thielen, P., and Salzberg, S.L. (2017). Bracken: estimating species abundance in metagenomics data. PeerJ Comput. Sci. *3*, e104. https://doi.org/10.7717/peerj-cs.104.

10. Menzel, P., Ng, K.L., and Krogh, A. (2016). Fast and sensitive taxonomic classification for metagenomics with Kaiju. Nat Commun *7*, 11257. https://doi.org/10.1038/ncomms11257.

11. Huerta-Cepas, J., Forslund, K., Coelho, L.P., Szklarczyk, D., Jensen, L.J., von Mering, C., and Bork, P. (2017). Fast Genome-Wide Functional Annotation through Orthology Assignment by eggNOG-Mapper. Molecular Biology and Evolution *34*, 2115–2122. https://doi.org/10.1093/molbev/msx148.
