# Supplementary material for: MOSHPIT: Accessible, reproducible metagenome data science on the QIIME 2 framework
Source: Cell Genom. 2026 Sep 24;6(10):101366. doi: 10.1016/j.xgen.2026.101366 (PMC13689502; doi:10.1016/j.xgen.2026.101366)
Supplement: Supplementary file 2 [file mmc2.pdf]

Cell Genomics

Technology

MOSHPIT: Accessible, reproducible metagenome data science on the QIIME 2 framework

Graphical abstract

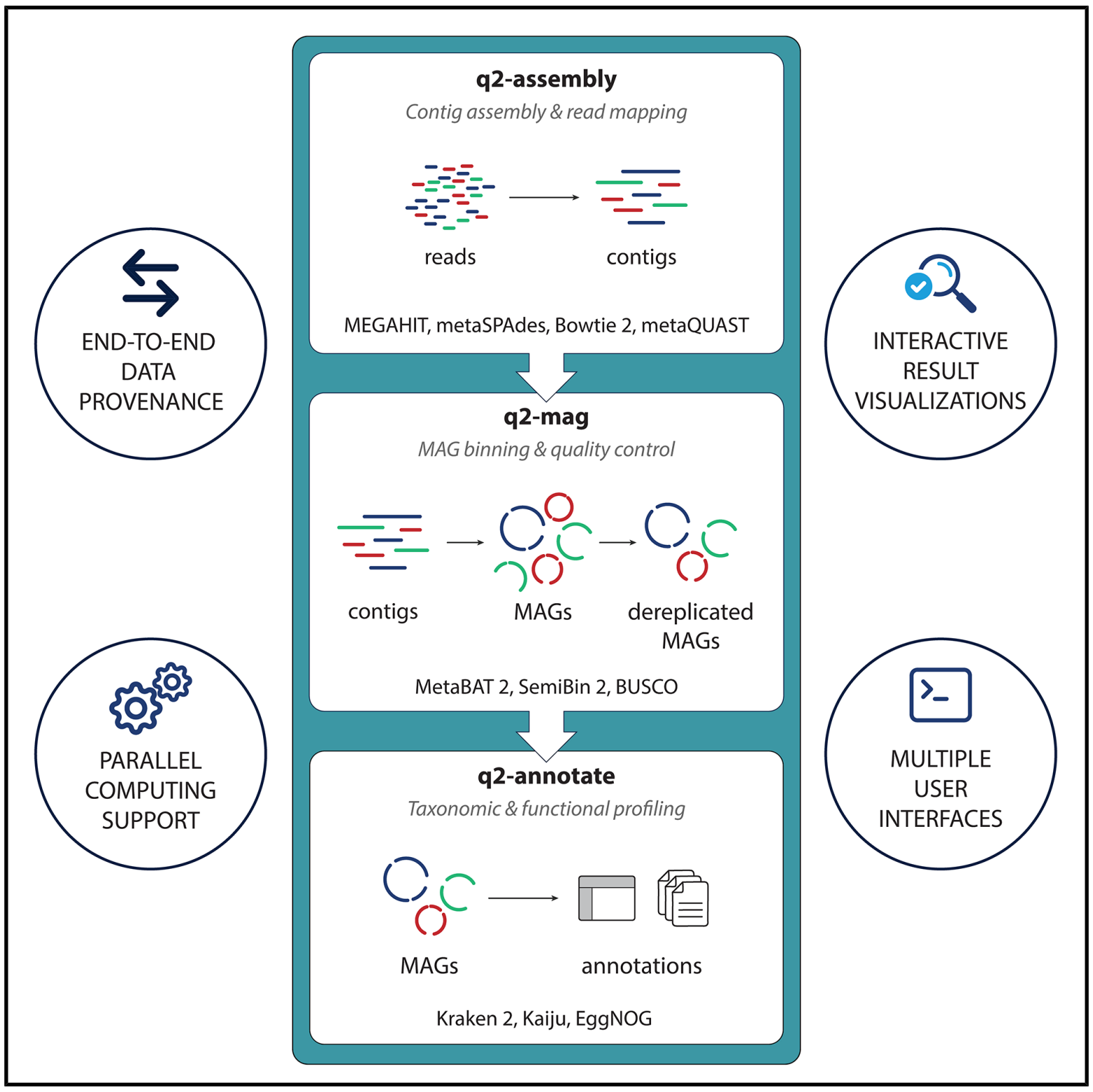

Authors

Michal Ziemski, Liz Gehret, Anthony Simard, ..., Evan Bolyen, J. Gregory Caporaso, Nicholas A. Bokulich

Correspondence

nicholas.bokulich@hest.ethz.ch

In brief

Ziemski et al. introduce MOSHPIT, a versatile software suite built on the QIIME 2 framework that simplifies complex shotgun metagenomic analysis. By integrating benchmarked tools into a unified, reproducible system based on the QIIME 2 framework with built-in data tracking, MOSHPIT democratizes microbial ecosystem research across diverse applications.

Highlights

- MOSHPIT brings reproducible shotgun metagenomics functionality to QIIME 2
- It integrates best-in-class benchmarked tools for assembly, binning, and annotation
- It ensures complete end-to-end data provenance and workflow scalability
- It supports read-, contig-, and MAG-based taxonomic and functional profiling

Ziemski et al., 2026, Cell Genomics 6, 101366
October 14, 2026 © 2026 The Author(s). Published by Elsevier Inc.
https://doi.org/10.1016/j.xgen.2026.101366

Cell Genomics

CellPress OPEN ACCESS

Technology

MOSHPIT: Accessible, reproducible metagenome data science on the QIIME 2 framework

Michal Ziemski,[1] Liz Gehret,[1] Anthony Simard,[2] Santiago Castro Dau,[1] Vinzent Risch,[1] Doriela Grabocka,[1] Christos Matzoros,[1] Colin Wood,[2] Paula Momo Cabrera,[1] Rodrigo Hernández-Velázquez,[1] Milo R. Schärer,[1] Felicia Sandberg,[1] Chloe Herman,[2] Keegan Evans,[2] Michael S. Robeson II,[3] Evan Bolyen,[1] J. Gregory Caporaso,[2,4] and Nicholas A. Bokulich[1,5,*]

[1]Department of Health Sciences and Technology, ETH Zurich, 8092 Zurich, Switzerland

[2]Pathogen and Microbiome Institute, Northern Arizona University, Flagstaff, AZ 86011, USA

[3]University of Arkansas for Medical Sciences, Department of Biomedical Informatics, Little Rock, AR 72205, USA

[4]Translational Genomics Research Institute, Flagstaff, AZ 86011, USA

[5]Lead contact

*Correspondence: nicholas.bokulich@hest.ethz.ch

https://doi.org/10.1016/j.xgen.2026.101366

SUMMARY

Metagenome sequencing has revolutionized functional microbiome analysis across diverse ecosystems but is fraught with technical hurdles. We introduce MOSHPIT (MOdular SHotgun metagenome Pipelines with Integrated provenance Tracking; https://moshpit.qiime2.org)—software built on the QIIME 2/rachis framework (Q2F) that integrates best-in-class CAMI II- and LEMMI-validated metagenome tools with robust provenance tracking and multiple user interfaces—enabling streamlined, reproducible metagenome analysis for all expertise levels. By building on Q2F, MOSHPIT enhances scalability, interoperability, and reproducibility in complex workflows, democratizing and accelerating discovery at the frontiers of metagenomics.

INTRODUCTION

Untargeted next-generation DNA sequencing has revolutionized microbiome research, yielding unparalleled insight into the composition, functionality, and interactions of microbial communities in diverse global ecosystems. The most common technique, marker-gene amplicon sequencing (e.g., of 16S rRNA genes), is widely used for its scalability and flexibility, but it provides a view of the microbial community that is contingent on PCR primer choice and enables only indirect inference of functional potential. Untargeted whole-metagenome (a.k.a., shotgun metagenome) sequencing (WMS) provides several advantages; it facilitates comprehensive community profiling of both functional gene and taxonomic composition; supports sub-species-level taxonomic resolution; and enables the assembly of metagenome-assembled genomes (MAGs) without the need for cultivation.[1] Numerous tools exist for different aspects of WMS analysis, but the complex workflows involved, lack of standardization, and issues with interoperability complicate analysis and hamper reproducibility.[2]

Since its official release in 2018,[3] the QIIME 2 microbiome bioinformatics platform has gained a wide user base for its accessibility, interoperability, and reproducibility. However, the QIIME 2/rachis framework (Q2F) and software ecosystem (https://rachis.org/) has historically lacked full support for untargeted WMS applications. Here, we introduce MOSHPIT (modular shotgun metagenome pipelines with integrated provenance tracking), a metagenomics software suite built on the Q2F supporting flexible, customizable, end-to-end WMS analysis (Figure 1A). MOSHPIT includes three core plugins and several optional accessory plugins that further enhance analysis (Figure 1B). The q2-assembly plugin (https://github.com/bokulich-lab/q2-assembly) provides a set of actions for contig assembly, evaluation, and read mapping; q2-mag (https://github.com/bokulich-lab/q2-mag) includes functionality for contig binning, quality control, and dereplication of the resulting MAGs, while q2-annotate (https://github.com/bokulich-lab/q2-annotate) supports downstream annotation steps for WMS data, including taxonomic and functional classification. Optional accessory plugins currently include q2-fastp (https://github.com/bokulich-lab/q2-fastp) for read trimming and quality control; q2-skani (https://github.com/bokulich-lab/q2-skani) for the average nucleotide identity (ANI) analysis; q2-gunc (https://github.com/bokulich-lab/q2-gunc) for MAG chimerism detection; q2-deseq2 (https://github.com/bokulich-lab/q2-deseq2) for differential expression/abundance analysis; q2-viromics (https://github.com/bokulich-lab/q2-viromics) and q2-virsorter2 (https://github.com/bokulich-lab/q2-virsorter2) for the detection and identification of viral sequences in WMS data; and q2-amrfinderplus (https://github.com/bokulich-lab/q2-amrfinderplus) and q2-rgi (https://github.com/bokulich-lab/q2-rgi) for the annotation of antimicrobial resistance genes in the assembled metagenomes via the popular AMRFinderPlus[4] and RGI/CARD tools.[5] To increase interoperability between these and other plugins, new data types, formats, and transformers have been added to the Q2F to facilitate streamlined analysis workflows for WMS datasets (https://github.com/qiime2/q2-types).

This is an open access article under the CC BY license (http://creativecommons.org/licenses/by/4.0/).

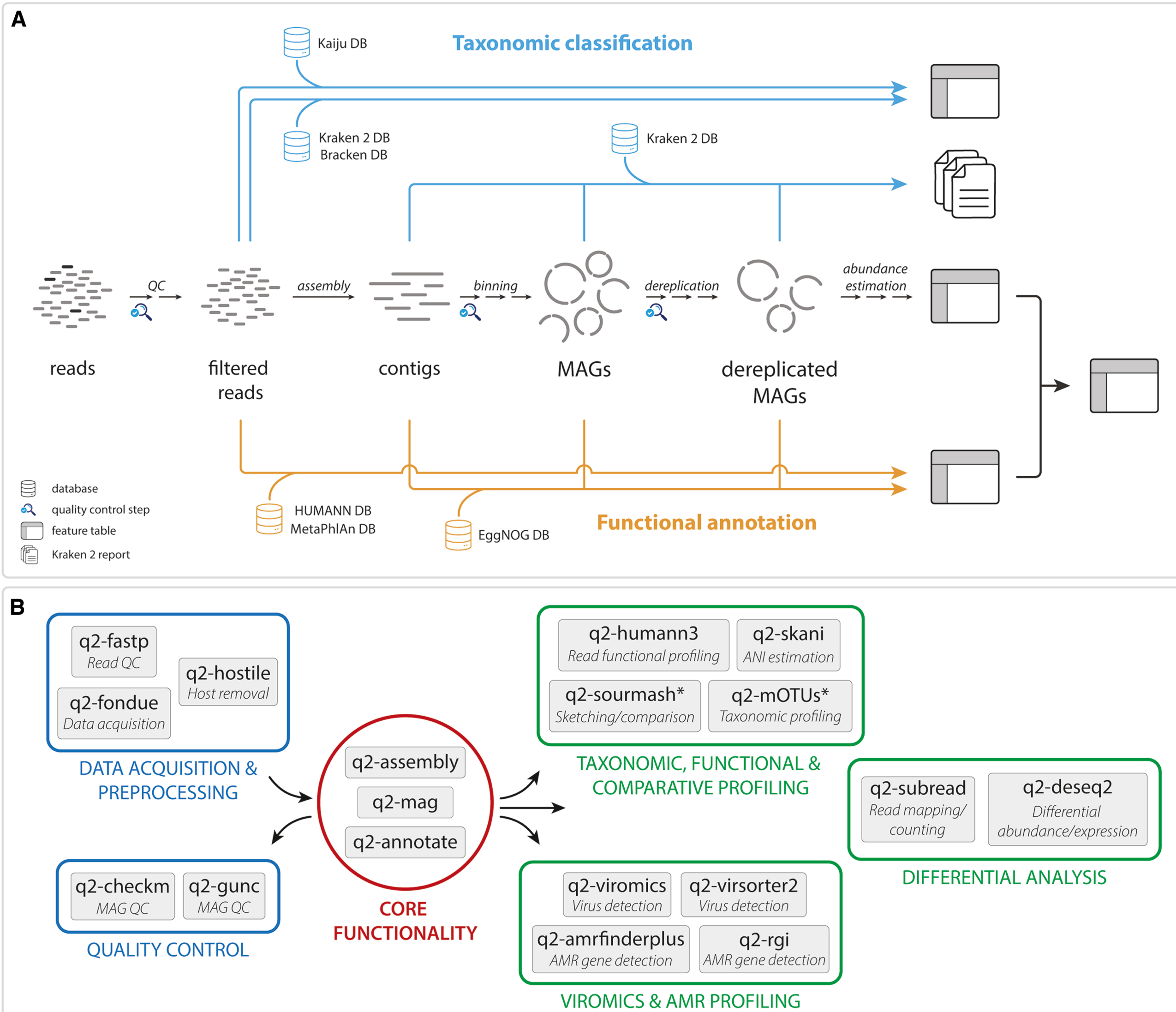

Figure 1. Schematic of current analysis workflows available in MOSHPIT and compatible plugins

(A) Overview of the major input/output types and analysis workflows available in MOSHPIT. Taxonomic annotation with Kaiju is supported for raw reads, and Kraken 2 can be applied to classify raw reads, contigs, or dereplicated MAGs. Functional annotation with EggNOG-mapper is available for contigs or (dereplicated) MAGs, while HUMANN can be used for read-based functional profiling.

(B) Overview of plugins compatible with the MOSHPIT workflows. The asterisk symbol (*) denotes the community plugins.

DESIGN

MOSHPIT integrates state-of-the-art tools selected from the top-performing methods in the CAMI II[6] and LEMMI[7] benchmarks and other methods to enable customizable, fully reproducible end-to-end WMS analysis workflows. This includes fastp for read quality control[8]; MEGAHIT[9] and metaSPAdes[10] for genome assembly; Bowtie 2[11] for read mapping; MetaBAT 2[12] for contig binning; QUAST[13] and BUSCO[14] for quality control of contigs and genomes; skani for ANI analysis[15]; GUNC for MAG chimerism detection[16]; and both read- and assembly-based taxonomic classification and functional annotation workflows via Kraken 2,[17] Kaiju,[18] and eggNOG-mapper[19] (see Figure 1 for an overview of the available workflows and plugins; see Figures S1–S4 for detailed sub-workflows).

MOSHPIT includes built-in support for retrieving diverse pre-built databases as well as constructing custom databases, e.g., for Kraken 2/Bracken, Kaiju, Diamond, and EggNOG. This integration streamlines workflows and enables precise versioning of reference databases used throughout the analysis, significantly enhancing reproducibility as database retrieval date, query parameters, and version information are embedded in provenance information.

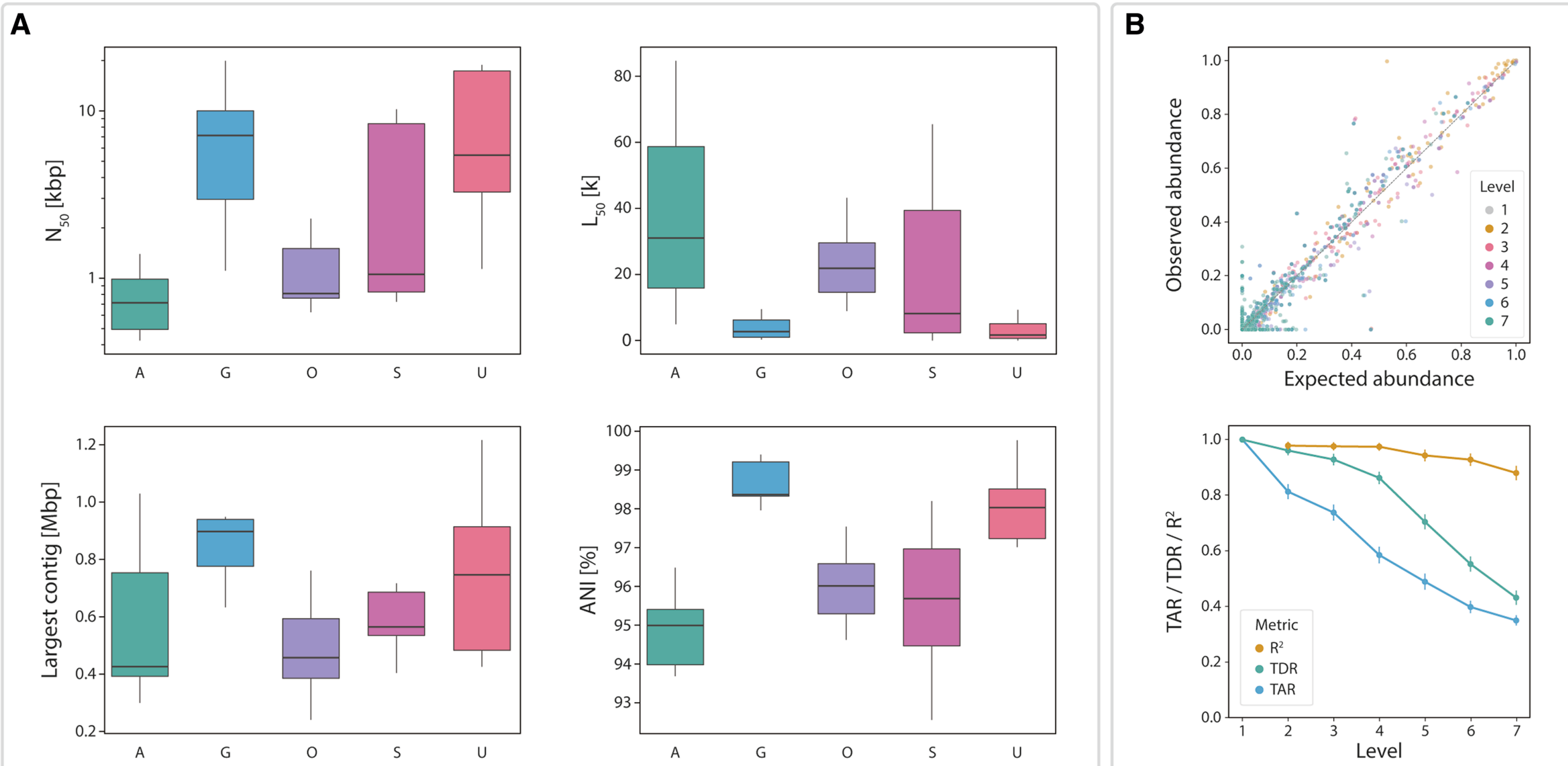

Figure 2. Analysis of the CAMI II toy human microbiome project dataset

(A) Assembly quality metrics obtained for all body sites (A: airways; G: gastrointestinal tract; O: oral cavity; S: skin; U: urogenital tract).

(B) Dereplicated MAG-based community recovery evaluation by comparison to the gold standard dataset. Error bars represent standard error of the mean.

Beyond the integration of existing tools, we have introduced custom actions to enhance MAG analysis, including MAG dereplication with q2-sourmash[20] (https://github.com/dib-lab/q2-sourmash) to produce a set of non-redundant genomes; estimating genome abundance through read mapping; integrating abundance data with functional annotation tables; and MAG quality filtering and human host read removal via mapping reads to the human pangenome[21] and GRCh38 genome (Figure S1). Customized host read filtering workflows, e.g., with other human or non-human reference genome collections, are also possible (through the "bowtie2-build" action from the q2-quality-control plugin).

Each workflow in MOSHPIT incorporates checkpoint visualizations, allowing users to inspect results throughout their analysis. Notable examples are: quality control of genome assembly (via the custom-built "evaluate-contigs" action or the "evaluate-quast" action incorporating metaQUAST[13]) and contig binning (via the "evaluate-busco" action incorporating BUSCO[14]), both of which are accessible via a web browser interface for interactive exploration and quality inspection.

MOSHPIT leverages recent enhancements to the Q2F to maximize efficiency in the analysis process. The artifact cache eliminates repeated compression and decompression of large QIIME Zipped Artifacts (.qza files), conserving time and computational resources. Additionally, built-in parallelization support, powered by Parsl,[22] enables users to process multiple data partitions simultaneously on systems ranging from multi-core laptops to high-performance compute clusters. Integrated pipeline resumption allows restarting interrupted or failed runs while reusing results that had already finished processing. Q2F's "provenance replay"[23] automates retrospective workflow documentation, facilitating transparency and replication.

RESULTS AND DISCUSSION

To benchmark the performance of a standard MOSHPIT pipeline configuration, we processed the CAMI II human microbiome toy dataset (49 samples, 16.6M reads each) and compared the resulting assemblies and MAGs to the provided gold standards (STAR Methods). Assembly continuity varied significantly by body site, with median largest contig lengths ranging from approximately 0.45 Mbp in the airways to 0.9 Mbp in the gastrointestinal tract (Figure 2A). We did not see a clear relationship between the assembly quality and the alpha diversity of the respective body sites (Figures 2A and S5). Despite variations in continuity, the assembly accuracy remained high, with the median contig ANI exceeding 95% across all body sites and reaching nearly 99% for gastrointestinal samples. MAG-based taxonomic profiling of the reconstructed communities further demonstrated strong correspondence to the ground truth (Figure 2B). Observed taxon abundances were strongly correlated with expected values, with the coefficient of determination (R^2) remaining above 0.9 through level 6 (family) and exceeding 0.85 at level 7 (genus). While the taxon accuracy rate (TAR) and taxon detection rate (TDR)[24] naturally declined at finer taxonomic resolutions, the pipeline maintained high precision in community reconstruction overall.

To demonstrate MOSHPIT's capability for handling large WMS datasets, we re-analyzed a subset of samples from the TARA Oceans Expedition,[25] specifically focusing on archaea

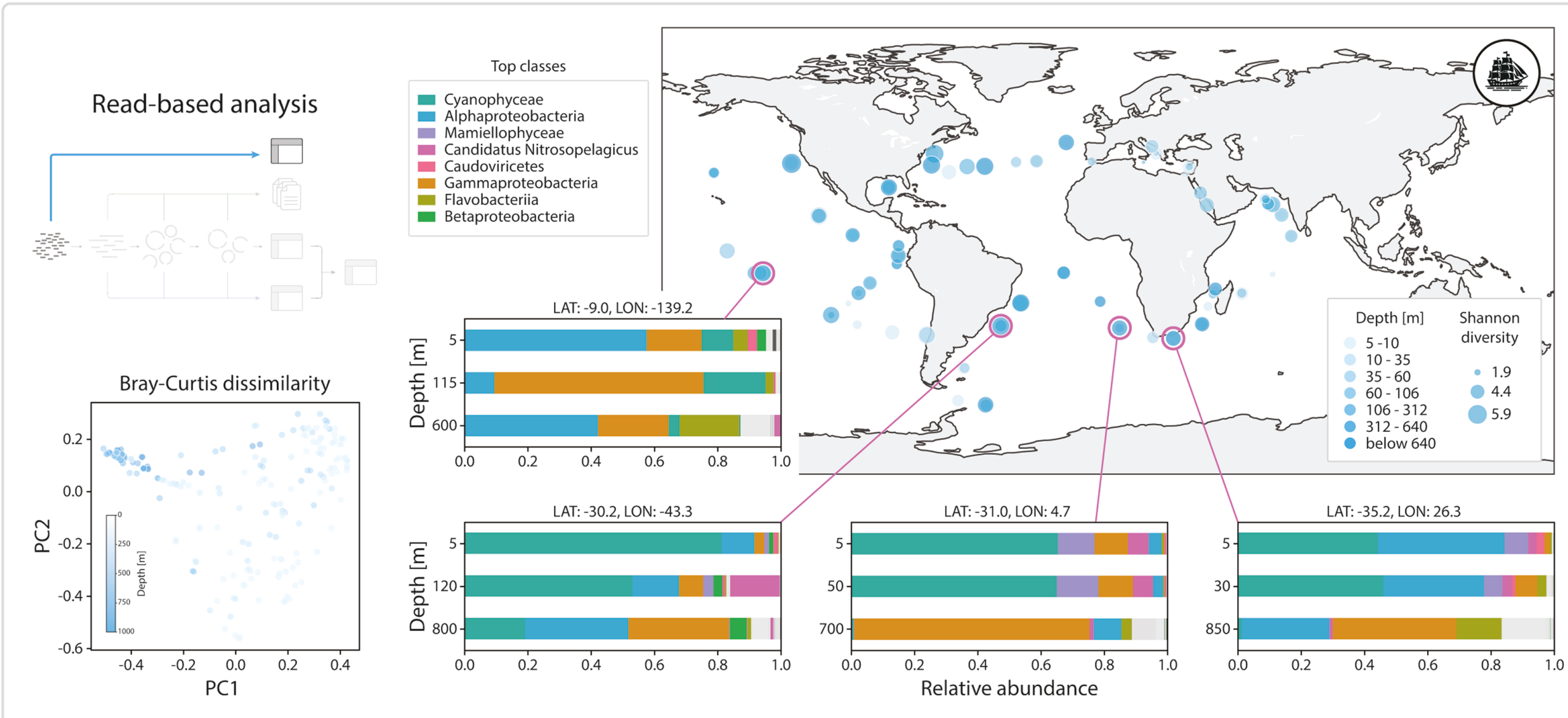

Figure 3. Reanalysis of the TARA Oceans dataset

The map depicts Shannon diversity of globally collected samples, with zoomed-in views of four locations showing taxonomic assignments across sample depths. Bray-Curtis principal coordinate scatterplot highlights compositional similarities among deep-sea samples.

and bacteria (STAR Methods). Leveraging MOSHPIT's interoperability with existing QIIME 2 plugins, we used q2-fondue[26] to retrieve original reads from the NCBI Sequence Read Archive (SRA), followed by taxonomic classification using Kraken 2 within q2-annotate and downstream diversity analysis in the Q2F (Figure 3).

To showcase a contig-based workflow using MOSHPIT plugins, we analyzed a set of 1,578 human gut microbiome samples from six different studies representing a gradient of industrialization including (subsistence) farmer, (subsistence) fisher, hunter-gatherer, industrialized rural, industrialized small city, and industrialized big city population (STAR Methods). Contigs were annotated with Kyoto Encyclopedia of Genes and Genomes (KEGG) KO terms, allowing the calculation of the per-sample abundance of each KEGG KO term. Permutational analysis of variance (PERMANOVA) showed that the population type was significantly associated with variance between samples (adonis2, $R^2 = 0.15$, $p = 0.001$), as reflected by sample clustering shown in Figure 4. ANCOM-BC2[27] was used to identify differentially abundant KEGG KO terms between industrialized big city and hunter gatherer populations. One of the KEGG KO terms with largest log fold changes between the two populations was K08169 (log-fold change = 3.57, $p < 0.001$, https://www.kegg.jp/entry/K08169),[28] which codes for a major facilitator superfamily transporter protein conferring multidrug resistance (Figure 4). This is also in line with previous studies indicating differences in the human gut resistome between the industrialized and non-industrialized populations.[29]

To demonstrate a MAG-based workflow and highlight compatibility with other community-supported plugins, we reanalyzed 14 cocoa fermentations[30] (STAR Methods). The results consistently recapitulate findings of the original study, via both

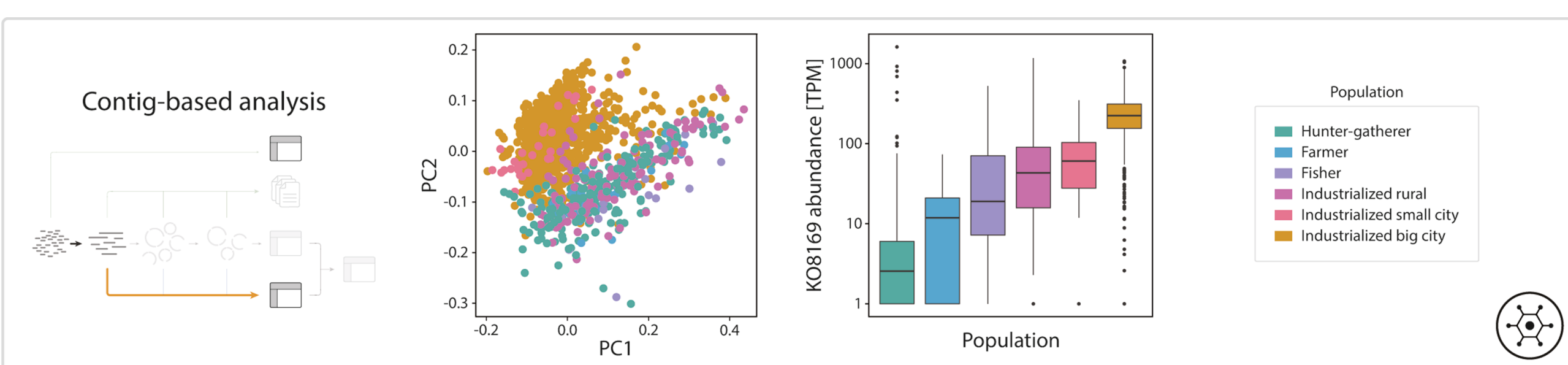

Figure 4. Analysis of the multi-study human microbiome

The principal coordinate scatterplot depicts sample grouping based on functional profiles from populations of the various urbanization levels. The box-whisker plots highlight a trend in the increase in abundance of a multidrug resistance transporter protein, K08169, with an increase in urbanization level.

CellPress
OPEN ACCESS

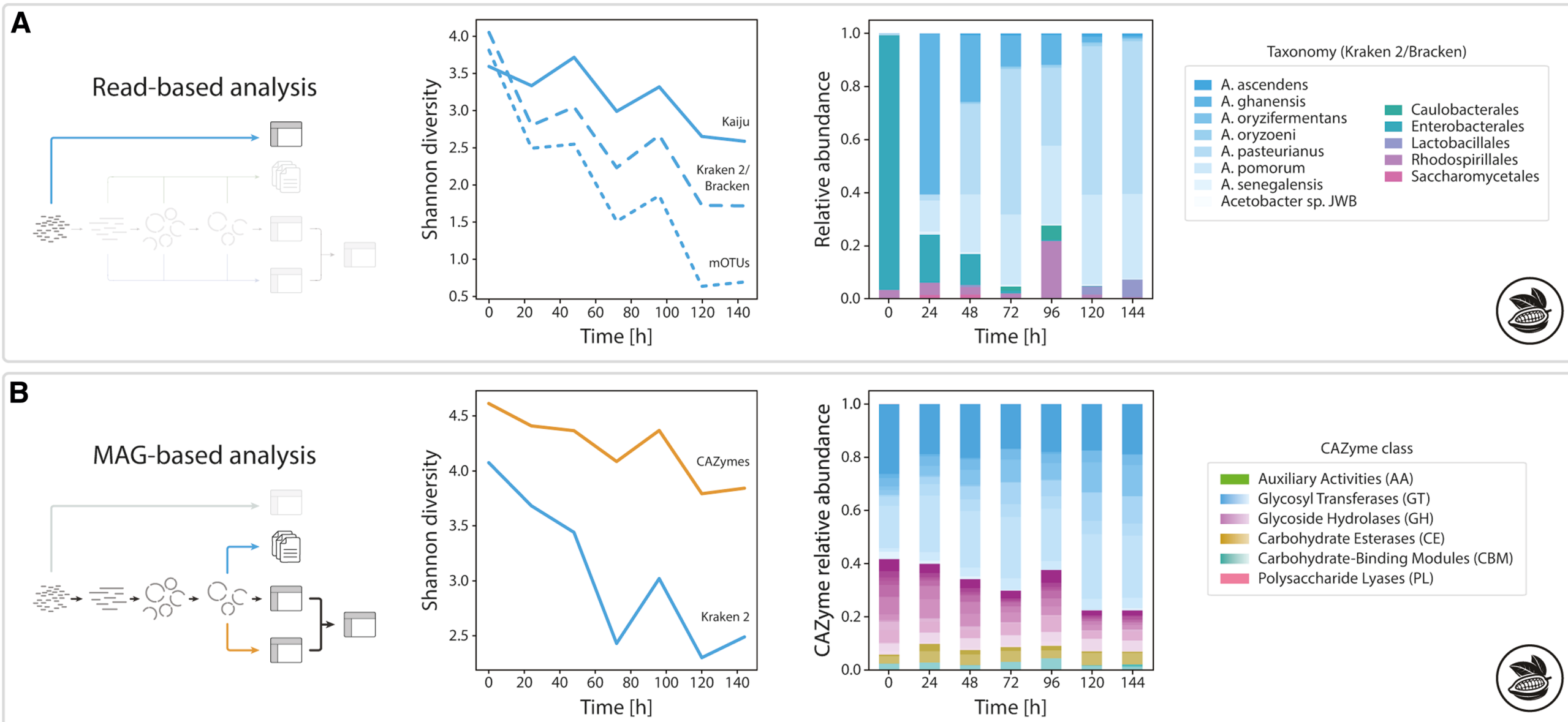

Figure 5. Reanalysis of the cocoa bean fermentation dataset

(A and B) Read-based (A) and MAG-based (B) analysis of cocoa demonstrates a consistent diversity decline over the fermentation process, accompanied by shifts in functional gene profile.

read-based classification with three methods (Kraken 2, Kaiju, and mOTUs 3, available via a community-developed MOSHPIT plugin) (Figure 5A) and MAG-based taxonomic classification and diversity analysis. The results obtained with all classifiers showed the decrease in taxonomic diversity during the cocoa fermentation process for both seed varieties (Figure 5A). Moreover, we could detect a significant decrease in abundance of the *Acetobacter* genus and a decrease in the *Pantoea* genus (Figure 5A), as reported by de C Lima et al.[30] Functional annotation of the recovered genomes in MOSHPIT yielded CAZyme gene counts for each sample (Figure 5B), revealing a decrease in CAZyme diversity over the course of the fermentation process, as determined by an increase in the abundance of CAZymes from the glycosyltransferase (GT) class and a decrease in enzymes classified as glycoside hydrolases (GH; Figure 5B).

MOSHPIT provides a comprehensive platform built on the Q2F to seamlessly integrate marker-gene and metagenome analyses, bridging a critical gap in the microbiome research community. By incorporating top-performing tools validated by benchmarks such as CAMI II and LEMMI for read-based and assembly-based workflows, alongside downstream tools for statistical analysis and visualization of diverse data types, MOSHPIT allows researchers to perform efficient, end-to-end analyses within a single, unified ecosystem, enabling flexible and fully reproducible integration of WMS analysis pipelines with the large variety of downstream analysis modules already available in QIIME 2. By wrapping diverse, top-performing utilities into the QIIME 2 ecosystem, MOSHPIT eliminates the need for manual, fragmented custom scripting across disparate tools. This enables seamless, provenance-tracked benchmarking and hybrid workflows, such as combining read-based profiling with downstream assembly and MAG binning tools, all within a single unified framework. Using the newly developed plugin development tutorial in "Developing with QIIME 2" (https://develop.qiime2.org), the ecosystem is readily extensible. Leveraging the Q2F's built-in parallelization features, users can now scale their workflows with ease, optimizing resource use across desktop and high-performance computing environments alike. This expansion enhances the accessibility, reproducibility, and scalability, while providing highly robust, benchmarked methods, thus capturing both taxonomic and functional dimensions of microbial communities for richer, more comprehensive community insights. As the landscape of methods for metagenome analysis evolves, we intend to expand MOSHPIT to include new tools. Within the next few release cycles, we will include additional binning and bin refinement tools, new taxonomic classifiers, and machine learning-based MAG quality assessment. Our upcoming roadmap also includes integrating biosynthetic gene cluster detection and dedicated support for eukaryotic metagenomics to keep pace with new developments in the field.

Limitations of the study

While MOSHPIT provides a standardized, reproducible framework for shotgun metagenomics within QIIME 2, several specific limitations of this software suite should be noted. First, to guarantee pipeline stability and maintainability, MOSHPIT selectively wraps a curated set of top-performing, CAMI II-validated algorithms. Consequently, alternative computational paradigms, such as long-read native assemblers or deep learning binners, are not yet natively wrapped in this core release at the time of publication. Second, standardizing standalone command-line tools into Q2F actions introduces interface abstractions that improve accessibility and automated provenance tracking but

slightly restrict niche parameter tuning compared with running standalone binaries directly. Third, while our empirical case studies validate end-to-end functionality across the ocean, gut, and food metagenomes, these demonstrations relied on public and/or subsetted datasets to showcase real-world utility, rather than providing exhaustive benchmarks across all possible biomes. Finally, storing large-scale metagenomic data within QIIME 2 artifact structures (.qza) requires managing disk storage overhead during caching and intermediate conversions when interfacing with non-QIIME 2 software. Ultimately, these design choices represent a deliberate balance between strict reproducibility and computational flexibility, establishing an extensible framework designed to adapt as metagenomic tools and community needs continue to evolve.

RESOURCE AVAILABILITY

Lead contact

Requests for further information and resources should be directed to and will be fulfilled by the lead contact, Nicholas A. Bokulich (nicholas.bokulich@hest.ethz.ch).

Materials availability

This study did not generate any new materials.

Data and code availability

- All the data used to demonstrate the functionality of the MOSHPIT software were fetched from the SRA using run IDs provided in the original studies. Provenance replay scripts detailing analysis of both datasets are provided in the https://github.com/bokulich-publications/moshpit-notebooks repository. The q2-assembly, q2-mag, and q2-annotate plugins are archived at Zenodo under their respective DOIs: https://doi.org/10.5281/zenodo.17703649, https://doi.org/10.5281/zenodo.22793586, and https://doi.org/10.5281/zenodo.17703645.
- Installation instructions, as well as user guides and tutorials demonstrating all the functionality described in this manuscript are available at https://moshpit.qiime2.org. These guides and tutorials will be regularly updated as new functionality is added to MOSHPIT.

ACKNOWLEDGMENTS

This work was supported, in part, by the Swiss National Science Foundation (grant 10008221 to N.A.B.); grant 2022-309890 from the Chan Zuckerberg Initiative DAF, an advised fund of the Silicon Valley Community Foundation, to N.A.B. and J.G.C.; by NIH National Cancer Institute Informatics Technology for Cancer Research award 1U24CA248454-01 to J.G.C.; and by the Open Research Data Program of the ETH Board to N.A.B.

AUTHOR CONTRIBUTIONS

M.Z., N.A.B., J.G.C., and E.B. designed the MOSHPIT software; M.Z., S.C.D., V.R., D.G., C.M., C.W., F.S., C.H., K.E., M.S.R., and N.A.B. contributed MOSHPIT code; L.G., A.S., C.W., and E.B. contributed necessary QIIME 2 framework code; M.Z., P.M.C., R.H.-V., M.R.S., C.H., and M.S.R. tested functionality of MOSHPIT; M.Z., P.M.C., R.H.-V., and N.A.B. wrote the usage tutorials; M.Z., N.A.B., J.G.C., and E.B. supervised the software development; N.A.B. and J.G.C. acquired funding support; M.Z. and N.A.B. wrote the manuscript; and L.G., J.G.C., and P.M.C. provided valuable comments on the manuscript. All authors have read and approved the final manuscript.

DECLARATION OF INTERESTS

J.G.C. and E.B. have equity in and are paid by Cymis Benefit Corporation.

STAR★METHODS

Detailed methods are provided in the online version of this paper and include the following:

- KEY RESOURCES TABLE
- METHOD DETAILS
  - Benchmarking
  - Case 1: TARA Oceans Expedition
  - Case 2: Multi-study human gut microbiome dataset
  - Case 3: Cocoa fermentation

SUPPLEMENTAL INFORMATION

Supplemental information can be found online at https://doi.org/10.1016/j.xgen.2026.101366.

Received: March 12, 2026
Revised: June 12, 2026
Accepted: September 1, 2026
Published: September 24, 2026

REFERENCES

1. Quince, C., Walker, A.W., Simpson, J.T., Loman, N.J., and Segata, N. (2017). Shotgun metagenomics, from sampling to analysis. Nat. Biotechnol. *35*, 833–844. https://doi.org/10.1038/nbt.3935.
2. Wooley, J.C., and Ye, Y. (2009). Metagenomics: Facts and artifacts, and computational challenges. J. Comput. Sci. Technol. *25*, 71–81. https://doi.org/10.1007/s11390-010-9306-4.
3. Bolyen, E., Rideout, J.R., Dillon, M.R., Bokulich, N.A., Abnet, C.C., Al-Ghalith, G.A., Alexander, H., Alm, E.J., Arumugam, M., Asnicar, F., et al. (2019). Reproducible, interactive, scalable and extensible microbiome data science using QIIME 2. Nat. Biotechnol. *37*, 852–857. https://doi.org/10.1038/s41587-019-0209-9.
4. Feldgarden, M., Brover, V., Gonzalez-Escalona, N., Frye, J.G., Haendiges, J., Haft, D.H., Hoffmann, M., Pettengill, J.B., Prasad, A.B., Tillman, G.E., et al. (2021). AMRFinderPlus and the Reference Gene Catalog facilitate examination of the genomic links among antimicrobial resistance, stress response, and virulence. Sci. Rep. *11*, 12728. https://doi.org/10.1038/s41598-021-91456-0.
5. Alcock, B.P., Huynh, W., Chalil, R., Smith, K.W., Raphenya, A.R., Wlodarski, M.A., Edalatmand, A., Petkau, A., Syed, S.A., Tsang, K.K., et al. (2023). CARD 2023: expanded curation, support for machine learning, and resistome prediction at the Comprehensive Antibiotic Resistance Database. Nucleic Acids Res. *51*, D690–D699. https://doi.org/10.1093/nar/gkac920.
6. Meyer, F., Fritz, A., Deng, Z.-L., Koslicki, D., Lesker, T.R., Gurevich, A., Robertson, G., Alser, M., Antipov, D., Beghini, F., et al. (2022). Critical Assessment of Metagenome Interpretation: the second round of challenges. Nat. Methods *19*, 429–440. https://doi.org/10.1038/s41592-022-01431-4.
7. Seppey, M., Manni, M., and Zdobnov, E.M. (2020). LEMMI: A continuous benchmarking platform for metagenomics classifiers. Genome Res. *30*, 1208–1216. https://doi.org/10.1101/gr.260398.119.
8. Chen, S., Zhou, Y., Chen, Y., and Gu, J. (2018). fastp: an ultra-fast all-in-one FASTQ preprocessor. Bioinformatics *34*, i884–i890. https://doi.org/10.1093/bioinformatics/bty560.
9. Li, D., Liu, C.M., Luo, R., Sadakane, K., and Lam, T.W. (2015). MEGAHIT: An ultra-fast single-node solution for large and complex metagenomics assembly via succinct de Bruijn graph. Bioinformatics *31*, 1674–1676. https://doi.org/10.1093/bioinformatics/btv033.
10. Nurk, S., Meleshko, D., Korobeynikov, A., and Pevzner, P.A. (2017). MetaSPAdes: A new versatile metagenomic assembler. Genome Res. *27*, 824–834. https://doi.org/10.1101/gr.213959.116.

11. Langmead, B., and Salzberg, S.L. (2012). Fast gapped-read alignment with Bowtie 2. Nat. Methods *9*, 357–359. https://doi.org/10.1038/nmeth.1923.

12. Kang, D.D., Li, F., Kirton, E., Thomas, A., Egan, R., An, H., and Wang, Z. (2019). MetaBAT 2: An adaptive binning algorithm for robust and efficient genome reconstruction from metagenome assemblies. PeerJ *7*, e7359. https://doi.org/10.7717/peerj.7359.

13. Mikheenko, A., Saveliev, V., and Gurevich, A. (2016). MetaQUAST: Evaluation of metagenome assemblies. Bioinformatics *32*, 1088–1090. https://doi.org/10.1093/bioinformatics/btv697.

14. Manni, M., Berkeley, M.R., Seppey, M., and Zdobnov, E.M. (2021). BUSCO: Assessing Genomic Data Quality and Beyond. Curr. Protoc. *1*, e323. https://doi.org/10.1002/cpz1.323.

15. (2023). Skani enables accurate and efficient genome comparison for modern metagenomic datasets. Nat. Methods *20*, 1633–1634. https://doi.org/10.1038/s41592-023-02019-2.

16. Orakov, A., Fullam, A., Coelho, L.P., Khedkar, S., Szklarczyk, D., Mende, D.R., Schmidt, T.S.B., and Bork, P. (2021). GUNC: detection of chimerism and contamination in prokaryotic genomes. Genome Biol. *22*, 178. https://doi.org/10.1186/s13059-021-02393-0.

17. Wood, D.E., Lu, J., and Langmead, B. (2019). Improved metagenomic analysis with Kraken 2. Genome Biol. *20*, 257. https://doi.org/10.1186/s13059-019-1891-0.

18. Menzel, P., Ng, K.L., and Krogh, A. (2016). Fast and sensitive taxonomic classification for metagenomics with Kaiju. Nat. Commun. *7*, 11257. https://doi.org/10.1038/ncomms11257.

19. Huerta-Cepas, J., Forslund, K., Coelho, L.P., Szklarczyk, D., Jensen, L.J., von Mering, C., and Bork, P. (2017). Fast Genome-Wide Functional Annotation through Orthology Assignment by eggNOG-Mapper. Mol. Biol. Evol. *34*, 2115–2122. https://doi.org/10.1093/molbev/msx148.

20. Pierce, N.T., Irber, L., Reiter, T., Brooks, P., and Brown, C.T. (2019). Large-scale sequence comparisons with sourmash. F1000Res. *8*, 1006. https://doi.org/10.12688/f1000research.19675.1.

21. Liao, W.-W., Asri, M., Ebler, J., Doerr, D., Haukness, M., Hickey, G., Lu, S., Lucas, J.K., Monlong, J., Abel, H.J., et al. (2023). A draft human pangenome reference. Nature *617*, 312–324. https://doi.org/10.1038/s41586-023-05896-x.

22. Babuji, Y., Woodard, A., Li, Z., Katz, D.S., Clifford, B., Kumar, R., Lacinski, L., Chard, R., Wozniak, J.M., Foster, I., et al. (2019). Parsl: Pervasive Parallel Programming in Python. Preprint at arXiv. https://doi.org/10.48550/arXiv.1905.02158.

23. Keefe, C.R., Dillon, M.R., Gehret, E., Herman, C., Jewell, M., Wood, C.V., Bolyen, E., and Caporaso, J.G. (2023). Facilitating bioinformatics reproducibility with QIIME 2 Provenance Replay. PLoS Comput. Biol. *19*, e1011676. https://doi.org/10.1371/journal.pcbi.1011676.

24. Bokulich, N.A., Kaehler, B.D., Rideout, J.R., Dillon, M., Bolyen, E., Knight, R., Huttley, G.A., and Gregory Caporaso, J. (2018). Optimizing taxonomic classification of marker-gene amplicon sequences with QIIME 2's q2-feature-classifier plugin. Microbiome *6*, 90. https://doi.org/10.1186/s40168-018-0470-z.

25. Sunagawa, S., Acinas, S.G., Bork, P., Bowler, C., Tara Oceans Coordinators; Eveillard, D., Gorsky, G., Guidi, L., Iudicone, D., Karsenti, E., et al. (2020). Tara Oceans: towards global ocean ecosystems biology. Nat. Rev. Microbiol. *18*, 428–445. https://doi.org/10.1038/s41579-020-0364-5.

26. Ziemski, M., Adamov, A., Kim, L., Flörl, L., and Bokulich, N.A. (2022). Reproducible acquisition, management and meta-analysis of nucleotide sequence (meta)data using q2-fondue. Bioinformatics *38*, 5081–5091. https://doi.org/10.1093/bioinformatics/btac639.

27. Lin, H., and Peddada, S.D. (2024). Multigroup analysis of compositions of microbiomes with covariate adjustments and repeated measures. Nat. Methods *21*, 83–91. https://doi.org/10.1038/s41592-023-02092-7.

28. Paradis, E., Claude, J., and Strimmer, K. (2004). APE: Analyses of Phylogenetics and Evolution in R language. Bioinformatics *20*, 289–290. https://doi.org/10.1093/bioinformatics/btg412.

29. Rampelli, S., Schnorr, S.L., Consolandi, C., Turroni, S., Severgnini, M., Peano, C., Brigidi, P., Crittenden, A.N., Henry, A.G., and Candela, M. (2015). Metagenome Sequencing of the Hadza Hunter-Gatherer Gut Microbiota. Curr. Biol. *25*, 1682–1693. https://doi.org/10.1016/j.cub.2015.04.055.

30. de C Lima, C.O., Vaz, A.B.M., De Castro, G.M., Lobo, F., Solar, R., Rodrigues, C., Martins Pinto, L.R., Vandenberghe, L., Pereira, G., Miúra da Costa, A., et al. (2021). Integrating microbial metagenomics and physicochemical parameters and a new perspective on starter culture for fine cocoa fermentation. Food Microbiol. *93*, 103608. https://doi.org/10.1016/j.fm.2020.103608.

31. Lu, J., Breitwieser, F.P., Thielen, P., and Salzberg, S.L. (2017). Bracken: estimating species abundance in metagenomics data. PeerJ Comput. Sci. *3*, e104. https://doi.org/10.7717/peerj-cs.104.

32. Asnicar, F., Berry, S.E., Valdes, A.M., Nguyen, L.H., Piccinno, G., Drew, D.A., Leeming, E., Gibson, R., Le Roy, C., Khatib, H.A., et al. (2021). Microbiome connections with host metabolism and habitual diet from 1,098 deeply phenotyped individuals. Nat. Med. *27*, 321–332. https://doi.org/10.1038/s41591-020-01183-8.

33. Carter, M.M., Olm, M.R., Merrill, B.D., Dahan, D., Tripathi, S., Spencer, S.P., Yu, F.B., Jain, S., Neff, N., Jha, A.R., et al. (2023). Ultra-deep sequencing of Hadza hunter-gatherers recovers vanishing gut microbes. Cell *186*, 3111–3124.e13. https://doi.org/10.1016/j.cell.2023.05.046.

34. Lokmer, A., Cian, A., Froment, A., Gantois, N., Viscogliosi, E., Chabé, M., and Ségurel, L. (2019). Use of shotgun metagenomics for the identification of protozoa in the gut microbiota of healthy individuals from worldwide populations with various industrialization levels. PLoS One *14*, e0211139. https://doi.org/10.1371/journal.pone.0211139.

35. Obregon-Tito, A.J., Tito, R.Y., Metcalf, J., Sankaranarayanan, K., Clemente, J.C., Ursell, L.K., Zech Xu, Z., Van Treuren, W., Knight, R., Gaffney, P.M., et al. (2015). Subsistence strategies in traditional societies distinguish gut microbiomes. Nat. Commun. *6*, 6505. https://doi.org/10.1038/ncomms7505.

36. Tamburini, F.B., Maghini, D., Oduaran, O.H., Brewster, R., Hulley, M.R., Sahibdeen, V., Norris, S.A., Tollman, S., Kahn, K., Wagner, R.G., et al. (2022). Short- and long-read metagenomics of urban and rural South African gut microbiomes reveal a transitional composition and undescribed taxa. Nat. Commun. *13*, 926. https://doi.org/10.1038/s41467-021-27917-x.

37. Cattaneo, A., Nelson, A., and McMenomy, T. (2021). Global mapping of urban-rural catchment areas reveals unequal access to services. Proc. Natl. Acad. Sci. USA *118*, e2011990118. https://doi.org/10.1073/pnas.2011990118.

38. McDonald, D., Clemente, J.C., Kuczynski, J., Rideout, J.R., Stombaugh, J., Wendel, D., Wilke, A., Huse, S., Hufnagle, J., Meyer, F., et al. (2012). The Biological Observation Matrix (BIOM) format or: how I learned to stop worrying and love the ome-ome. GigaScience *1*, 7. https://doi.org/10.1186/2047-217X-1-7.

39. Wickham, H., Averick, M., Bryan, J., Chang, W., McGowan, L., François, R., Grolemund, G., Hayes, A., Henry, L., Hester, J., et al. (2019). Welcome to the Tidyverse. J. Open Source Softw. *4*, 1686. https://doi.org/10.21105/joss.01686.

40. Dixon, P. (2003). VEGAN, a package of R functions for community ecology. J. Veg. Sci. *14*, 927–930. https://doi.org/10.1111/j.1654-1103.2003.tb02228.x.

41. Ruscheweyh, H.-J., Milanese, A., Paoli, L., Karcher, N., Clayssen, Q., Keller, M.I., Wirbel, J., Bork, P., Mende, D.R., Zeller, G., and Sunagawa, S. (2022). Cultivation-independent genomes greatly expand taxonomic-profiling capabilities of mOTUs across various environments. Microbiome *10*, 212. https://doi.org/10.1186/s40168-022-01410-z.

STAR★METHODS

KEY RESOURCES TABLE

REAGENT or RESOURCE	SOURCE	IDENTIFIER
Software and algorithms		
Fastp	Chen et al.[8]	https://github.com/opengene/fastp
MEGAHIT	Li et al.[9]	https://github.com/voutcn/MEGAHIT
metaSPAdes	Nurk et al.[10]	https://github.com/ablab/spades
Bowtie 2	Langmead et al.[11]	https://github.com/benlangmead/bowtie2
MetaBAT 2	Kang et al.[12]	https://bitbucket.org/berkeleylab/metabat/src/master/
QUAST	Mikheenko et al.[13]	https://github.com/ablab/quast
BUSCO	Manni et al.[14]	https://gitlab.com/ezlab/busco
Skani	Shaw et al.[15]	https://github.com/bluenote-1577/skani
GUNC	Orakov et al.[16]	https://github.com/grp-bork/gunc
Kraken 2	Wood et al.[17]	https://github.com/DerrickWood/kraken2
Bracken	Lu et al.[31]	https://github.com/jenniferlu717/Bracken
Kaiju	Menzel et al.[18]	https://github.com/bioinformatics-centre/kaiju
EggNOG-mapper	Huerta-Cepas et al.[19]	https://github.com/eggnogdb/eggnog-mapper
SourMASH	Pierce et al.[20]	https://github.com/sourmash-bio/sourmash
Deposited data		
Human reference genome, GRCh38	Genome Reference Consortium	https://www.ncbi.nlm.nih.gov/datasets/genome/GCF_000001405.40/
Human pangenome	Human Pangenome Reference Consortium	ftp://ftp.sra.ebi.ac.uk/vol1/analysis/ERZ127/ERZ12792464/hprc-v1.0-pggb.gfa.gz
CAMI II Toy Human Microbiome Project	Meyer et al.[6]	https://frl.publisso.de/data/frl:6425518/
TARA Oceans	Sunagawa et al.[25]	https://www.ncbi.nlm.nih.gov/bioproject/173486
Cocoa fermentation	de C Lima et al.[30]	https://www.ncbi.nlm.nih.gov/bioproject/552479

METHOD DETAILS

Benchmarking

Data retrieval and quality control

We used the CAMI II[6] Toy Human Microbiome Project (THMP) dataset to ensure validity of the results generated by the analysis pipeline constructed using only plugins from the MOSHPIT distribution. Simulated reads for 49 samples from the THMP dataset were fetched using the camiClient, as per the CAMI II challenge instructions (https://cami-challenge.org/datasets/Toy%20Human%20Microbiome%20Project/). The golden standard genomes and OTU abundances were obtained in a similar fashion.

The reads underwent a quality control step using the *process-seqs* action from the q2-fastp plugin (*qualify_quality_phred* = 30, *cut_tail* = true; https://github.com/bokulich-lab/q2-fastp). Subsequently, the contaminating reads of human origin were removed using the *filter-reads-pangenome* action from the q2-annotate plugin (https://github.com/bokulich-lab/q2-annotate). The read depth of all the samples across all body sites was 16.6 M.

MAG reconstruction and annotation

Reads were assembled into contigs using the metaSPAdes[10] assembler through the *assemble-spades* action from the q2-assembly plugin with the "meta" preset. The contigs were indexed with Bowtie 2[11] (*index-contigs* action) and the reads were mapped to contigs using those indices as input to the *map-reads* action.

Contig binning was performed with MetaBAT 2[12] through the *bin-contigs-metabat* action from the q2-annotate plugin. The resulting MAGs were subject to quality control with BUSCO.[14] The database of prokaryotic BUSCOs (*bacteria_odb12*) was fetched using the *fetch-busco-db* action and used as input (together with the generated MAGs) to the *evaluate-busco* action.

The resulting QC table was used to filter the MAGs based on the completeness score - only bacterial MAGs which were at least 50% complete were retained (*filter-mags* action from q2-annotate with *on* = mag and *where* = "completeness>50″ parameters).

To generate a non-redundant set of MAGs we developed a dereplication action (*dereplicate-mags*) which uses a distance matrix of the original MAGs, clusters similar MAGs given a similarity threshold and identifies the single MAG representative of each cluster

CellPress
OPEN ACCESS

(based on the assembly length). The distance matrix was obtained using the SourMash[20] tool: MinHash signatures of each MAG were calculated using the *compute* action from the sourmash plugin (using *ksizes* = 35 and *scaled* = 10 parameters) and compared using the *compare* action from the same plugin. The resulting distance matrix was then used as input to the *dereplicate-mags* action which generated a non-redundant set of MAGs at the similarity threshold of 0.97.

The taxonomic identity of the dereplicated MAGs was determined using the *classify-kraken2* action as described in the section below (Tara Oceans dataset analysis). The taxonomic assignment of each MAG was then obtained through the *kraken2-to-mag-features* action which performs an LCA selection from all the classified contigs within each individual MAG.

To obtain the abundances of each MAG in the original community we developed a set of actions which map the original reads to the dereplicated MAGs and use read counts (expressed as RPKM or TPM values) as a proxy for abundance. We first indexed the dereplicated MAGs using the *index-derep-mags* action (q2-assembly), followed by mapping the reads to the generated indices using the *map-reads* action. The obtained alignment maps were then used as input to the *estimate-abundance* action (together with MAG lengths extracted using the *get-feature-lengths* action). In order to only retain high-quality mapped reads we set the *min_mapq* parameter to 42 and *min_base_quality* to 20. This action uses the *samtools coverage* tool to extract coverage of each MAG and uses the extracted counts to estimate the RPKM and TPM values.

Evaluation

To evaluate the quality of the assembled metagenomes we ran metaQUAST[13] by providing the golden standard genomes as the reference.

We used the mapping files provided together with the golden standard assembly data to reconstruct feature tables for each body site. Those feature tables were then used as input to the *evaluate-composition* action from the q2-quality-control plugin (https://github.com/qiime2/q2-quality-control) which provided all the reported metrics (TAR, TDR, R^2).

To evaluate whether the assembly quality correlated with the body site diversity we performed taxonomic classification using Kraken 2 as described in the sections below. The observed feature count as well as Shannon diversity metrics were obtained by running the *core-metrics* action from the q2-diversity plugin using a sampling depth of 16′000′000.

Case 1: TARA Oceans Expedition

Data retrieval and quality control

Sequencing data for 243 samples from the PRJEB1787 BioProject (SRA: PRJEB1787) that correspond to the prokaryotic fraction of the ocean water was fetched from the SRA archive using the q2-fondue plugin (https://github.com/bokulich-lab/q2-fondue) using the default parameters.

To ensure high-quality of read classification, reads were filtered using the q2-cutadapt plugin (*trim-paired* action) to remove all the reads shorter than 90 bp (*minimum_length* = 90).

Read-based analysis

Read-based taxonomic classification was performed using the Kraken 2[17] tool built into the q2-annotate plugin. The Kraken 2 PlusPFP database was fetched using the *build-kraken-db* action and was used to classify the reads through the *classify-kraken2* action using the confidence score of 0.2 (while keeping all the other parameters at their default values). Following the classification, read counts were corrected for genome lengths using Bracken[31] through the *estimate-bracken* action (with *read_len* = 100 and *threshold* = 5 parameters) using the respective database fetched together with the Kraken 2 PlusPFP (see above). Subsequently, any unclassified reads or reads classified as *Homo sapiens* were removed from the resulting feature table by using the *filter-table* action from the feature-table plugin. Alpha- and beta-diversity metrics were calculated using the *core-metrics* action from the q2-diversity plugin using a sampling depth of 617′000.

Case 2: Multi-study human gut microbiome dataset

Data retrieval, quality control and sample metadata

A collection of 1578 manually curated samples from six different studies[29,32–36] were retrieved from SRA using the *get-sequences* action from the q2-fondue plugin (https://github.com/bokulich-lab/q2-fondue). Due to resource limitations on the high performance cluster on which the analysis was performed, fetching and processing up to feature tables was done in batches of 15–30 samples. Samples were categorized as farmer, fisher, hunter-gatherer or industrialized as described by Lokmer et al.[34] Based on latitude/longitude metadata retrieved using the *get-metadata* action from q2-fondue, samples were further assigned a value from 1 (most urbanized) to 30 (least urbanized) according to the urban-rural catchment areas (URCA) method.[37] Industrialized samples with an URCA value less than 3 were classified as "industrialized big city", those with a value greater than or equal to 3 but less than 12 were classified as "industrialized small city", those with a value of 12 or more were classified as "industrialized rural". Quality control was done using the *process-seqs* action (*qualified_quality_phred* = 30) from the q2-fastp plugin (https://github.com/bokulich-lab/q2-fastp). Contamination from human host reads was removed using the *filter-reads-pangenome* action from the q2-annotate plugin.

Contig assembly and annotation

Processed reads were assembled into contigs using the *assemble-megahit* action from the q2-assembly plugin with the preset 'meta-sensitive'. Protein orthologs within contigs were found using the *search-orthologs-diamond* action and orthologs were annotated with KEGG KO terms using the *map-eggnog* action, both from q2-annotate. The q2-annotate *extract-annotations* action was used to generate a feature table of KEGG KO terms per contig. The *map-reads* action from q2-assembly was used to map reads back

to contigs, allowing the generation of contig per sample abundance feature tables with the q2-annotate actions *get-feature-lengths* and *estimate-abundance* (*min_mapq* = 42, *metric* = tpm). The *multiply-tables* action from q2-feature-table was used to generate feature tables of KEGG KO terms per sample. The *merge-tables* action from q2-feature-table was used to merge feature tables from all sample processing batches into one.

Statistical analysis and plotting

The feature table of KEGG KO terms per sample was exported from qiime2 using the *export* action from the tool suite of the Q2 framework (https://github.com/qiime2/q2cli/blob/dev/q2cli/builtin/tools.py) and converted from biom to TSV format.[38] Data analysis and plotting was done in R (version 4.5.2) within the R studio integrated development environment (IDE) (version 2026.01.1 + 403) with the packages ape (version 5.8.1[28]), janitor (version 2.21, https://github.com/sfirke/janitor), scales (version 1.4.0, https://scales.r-lib.org), tidyverse (version 2.0.0[39]), vegan (version 2.7–2[40]) and viridis (0.6.5, https://zenodo.org/records/7890878). Principal coordinate analysis was performed on a pairwise Euclidean sample distance matrix of a Hellinger-transformed feature table. Based on differential abundance analysis performed on the feature table using AncomBC 2 from the q2-composition plugin, a KEGG KO term with a large fold change between industrialized big city and hunter-gatherer samples was selected for plotting in R.

Case 3: Cocoa fermentation

Data retrieval

Sequencing data for all 14 time-resolved shotgun metagenomics samples obtained for two different varieties of cocoa beans (7 samples per variety) from the BioProject PRJNA552479 (SRA: PRJNA552479) was fetched from the SRA archive using the q2-fondue plugin (https://github.com/bokulich-lab/q2-fondue) using the default parameters.

Read-based analysis

Read-based taxonomic classification was performed using three different classifiers available through QIIME 2 plugins. Kraken 2/Bracken and Kaiju classifiers are available as part of the MOSHPIT suite (within the q2-annotate plugin; https://github.com/bokulich-lab/q2-annotate) and mOTUs 3[41] is available as a community plugin (https://github.com/motu-tool/q2-mOTUs).

The Kraken 2 PlusPF database was fetched using the *build-kraken-db* action from the q2-annotate plugin. It was then used to classify the reads through the *classify-kraken2* action using a confidence score of 0.5 (while keeping all the other parameters at their default values). Following the classification, read counts were corrected for genome lengths using Bracken through the *estimate-bracken* action (with *read_len* = 150 and *threshold* = 5 parameters) using the respective database fetched together with the Kraken 2 PlusPF (see above).

The mOTUs classification was performed using the default parameters through the *profile* action from the mOTUs plugin.

The nr_euk Kaiju database was fetched using the *fetch-kaiju-db* action from the q2-annotate plugin and used to classify the reads with the *classify-kaiju* action (with *c* = 0.1).

Unclassified reads were removed from the resulting feature tables by using the *filter-table* action from the q2-feature-table plugin. Alpha- and beta-diversity metrics were calculated using the *core-metrics* action from the q2-diversity plugin using sampling depths of 2′320′000 (for Kraken 2/Bracken), 5′298′000 (for Kaiju) or 1′792 (for mOTUs).

MAG-based analysis

Reads were assembled into contigs using the MEGAHIT[9] assembler through the *assemble-megahit* action from the q2-assembly plugin with the "meta-sensitive" preset. The quality of the generated assemblies was checked with metaQUAST[13] through the *evaluate-contigs* action. The contigs were indexed with Bowtie 2[11] (*index-contigs* action) and the reads were mapped to contigs using those indices as input to the *map-reads* action.

Contig binning, filtering and dereplication of the resulting bins were performed as described above in the "Toy Human Microbiome Project dataset benchmark" section. The similarity threshold used during dereplication was set to 0.99.

The taxonomic identity of the dereplicated MAGs was determined using the *classify-kraken2* action as described above in the section "TARA Oceans dataset analysis". The taxonomic assignment of each MAG was then obtained through the *kraken2-to-mag-features* action which performs an LCA selection from all the classified contigs within each individual MAG.

The MAG abundances were estimated as described in the "Toy Human Microbiome Project dataset benchmark" section.

The functional profile of each community was obtained using the EggNOG[19] annotation tool which we incorporated into the q2-annotate plugin. The required databases were retrieved using the *fetch-diamond-db* and *fetch-eggnog-db* actions. The ortholog candidates were identified using the *search-orthologs-diamond* action and the dereplicated set of MAGs. The resulting ortholog tables were then input to the *map-eggnog* action (together with the corresponding database) which generated tables of EggNOG annotations. We used the *extract-annotations* action to retrieve the CAZyme annotations from the tables obtained from EggNOG and convert them into a feature table (using *annotation* = caz and *max_evalue* = 0.0001 parameters). This feature table was then input to the *multiply-tables* action (together with the MAG abundance feature table) to calculate their dot product representing abundances of each CAZyme in each sample.

Distribution and installation

The MOSHPIT software suite is provided as a self-contained QIIME 2 distribution named "moshpit", in the form of fully resolved conda environments and docker images. All the updates to any of the MOSHPIT plugins will follow the regular QIIME 2 release cycle

CellPress
OPEN ACCESS

and will be announced on the QIIME 2 forum (https://forum.qiime2.org). All the information about the exact plugin composition and installation instructions for all the supported operating systems can be found in the official QIIME 2 documentation on https://library.qiime2.org.

MOSHPIT nextflow workflow

To enable standardized end-to-end analyses of whole metagenome data using MOSHPIT we also provide a Nextflow workflow which allows deployment of all of the described functionality on a variety of clusters and computing environments using both, conda environments and containers. The workflow can be found under https://github.com/bokulich-lab/nf-moshpit.

Cell Genomics, Volume *6*

Supplemental information

MOSHPIT: Accessible, reproducible

metagenome data science on the QIIME 2 framework

Michal Ziemski, Liz Gehret, Anthony Simard, Santiago Castro Dau, Vinzent Risch, Doriela Grabocka, Christos Matzoros, Colin Wood, Paula Momo Cabrera, Rodrigo Hernández-Velázquez, Milo R. Schärer, Felicia Sandberg, Chloe Herman, Keegan Evans, Michael S. Robeson II, Evan Bolyen, J. Gregory Caporaso, and Nicholas A. Bokulich

Supplemental Figures

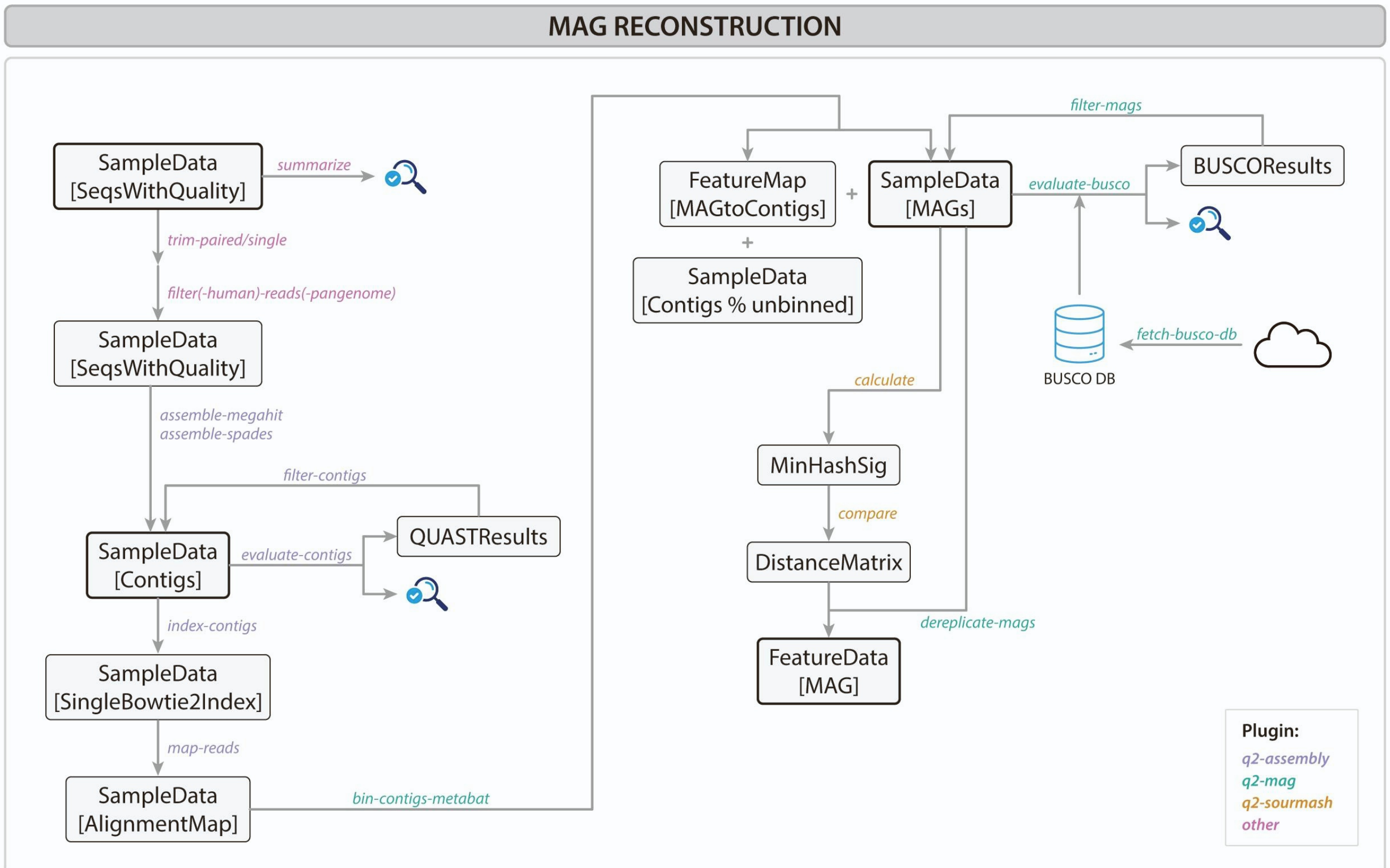

Figure S1: MOSHPIT MAG reconstruction workflow, related to Figure 1A. MAG reconstruction workflow comprises all the steps which are required to recover Metagenome-Assembled Genomes from reads, including their dereplication into a non-redundant set of genomes. Initially, reads can be quality controlled/filtered to remove potential contaminants (e.g. host reads). On top of the *filter-reads* action (available through the existing q2-quality-control plugin), we developed the new *filter-reads-human-pangenome* action (as part of the q2-quality-control plugin) which allows users to automatically construct an index comprised of the GRCh38 reference human genome and the most recently published human pangenome[1] and use it to filter out human host reads. Following the quality control steps, metagenome assembly can be performed using one of the two actions: *assemble-megahit* or *assemble-spades*, which will use the respective assembler (MEGAHIT[2] or SPAdes[3]) to reconstruct the contigs. Those can then be quality controlled using the *evaluate-quast* [4] or *evaluate-contigs* actions and indexed with the *index-contigs* action. In preparation for the binning step, the original reads can then be mapped to the contigs (*map-reads* action) using the index generated in the previous step. These contigs are then used as an input to the *bin-contigs-metabat* action, which will use MetaBat 2[5] to form the bins. Those can then be quality controlled with the *evaluate-busco* action[6]. Following binning, construction of a non-redundant set of genomes may be performed by first generating a distance matrix for the given genome set and using it to cluster genomes together, selecting one representative per cluster. Our workflow makes use of the per-MAG MinHash signatures generated through the *calculate* action (q2-sourmash plugin[7]), followed by the *compare* action (from the same plugin) to generate the distance matrix. The resulting table can then be used as input to our *dereplicate-mags* action which will find MAG clusters based on a distance threshold using hierarchical clustering with Ward linkage. It will then select the longest/most complete genome in each cluster (depending on whether completeness information was provided) to be its representative and return those non-redundant genomes together with a presence-absence table indicating which samples they were found in. Finally, to estimate the abundance of those genomes in the original samples, the dereplicated MAGs

can be indexed using the *index-derep-mags* action (q2-assembly), followed by read mapping using the *map-reads* action. The resulting maps, together with genome lengths (obtained via the *get-feature-lengths* action), can be input into the *estimate-abundance* action. This action will count the reads mapped to every MAG (while applying filtering parameters like minimal mapping quality, read length or base quality) and convert the counts using one of the available metrics like RPKM or TPM. Those metrics will then be returned as a feature table which can be used with any of the existing downstream QIIME 2 actions.

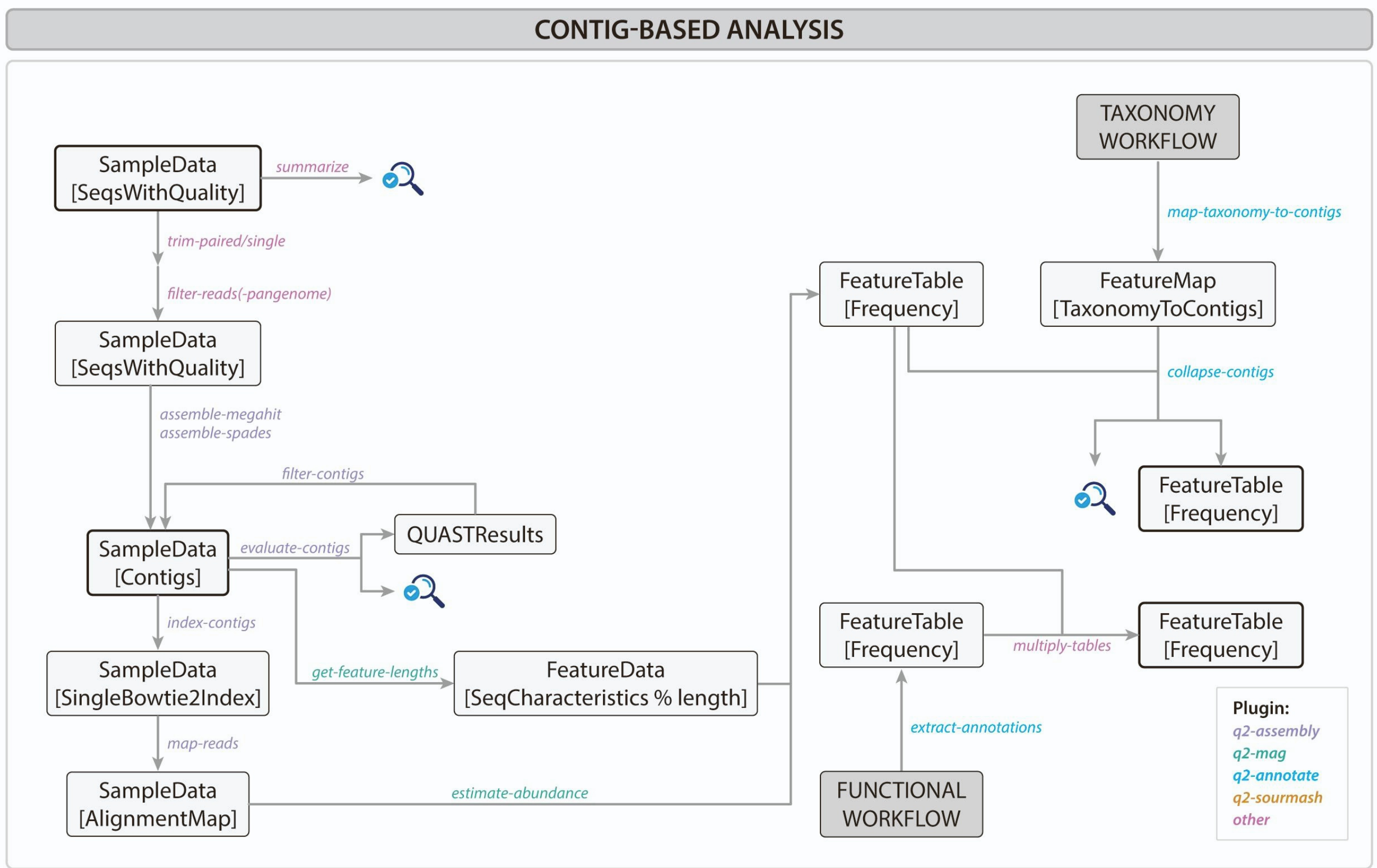

Figure S2: MOSHPIT contig-based analysis workflow, related to Figure 1A. Contig-based analysis workflow provides insight into community composition and functional profile already at the stage of genome assembly, without the need for contig binning and MAG reconstruction. The *estimate-abundance* action provides abundance estimates for each contig in a given sample and, when followed by the *collapse-contigs* action, can provide an early glimpse into the taxonomic composition of each sample. Additionally, functional profiles can be obtained by annotating contigs using one of the *search-orthologs-diamond* or *search-orthologs-hmmer* actions, followed by *map-eggnog*, *extract-annotations* and *multiply-tables*.

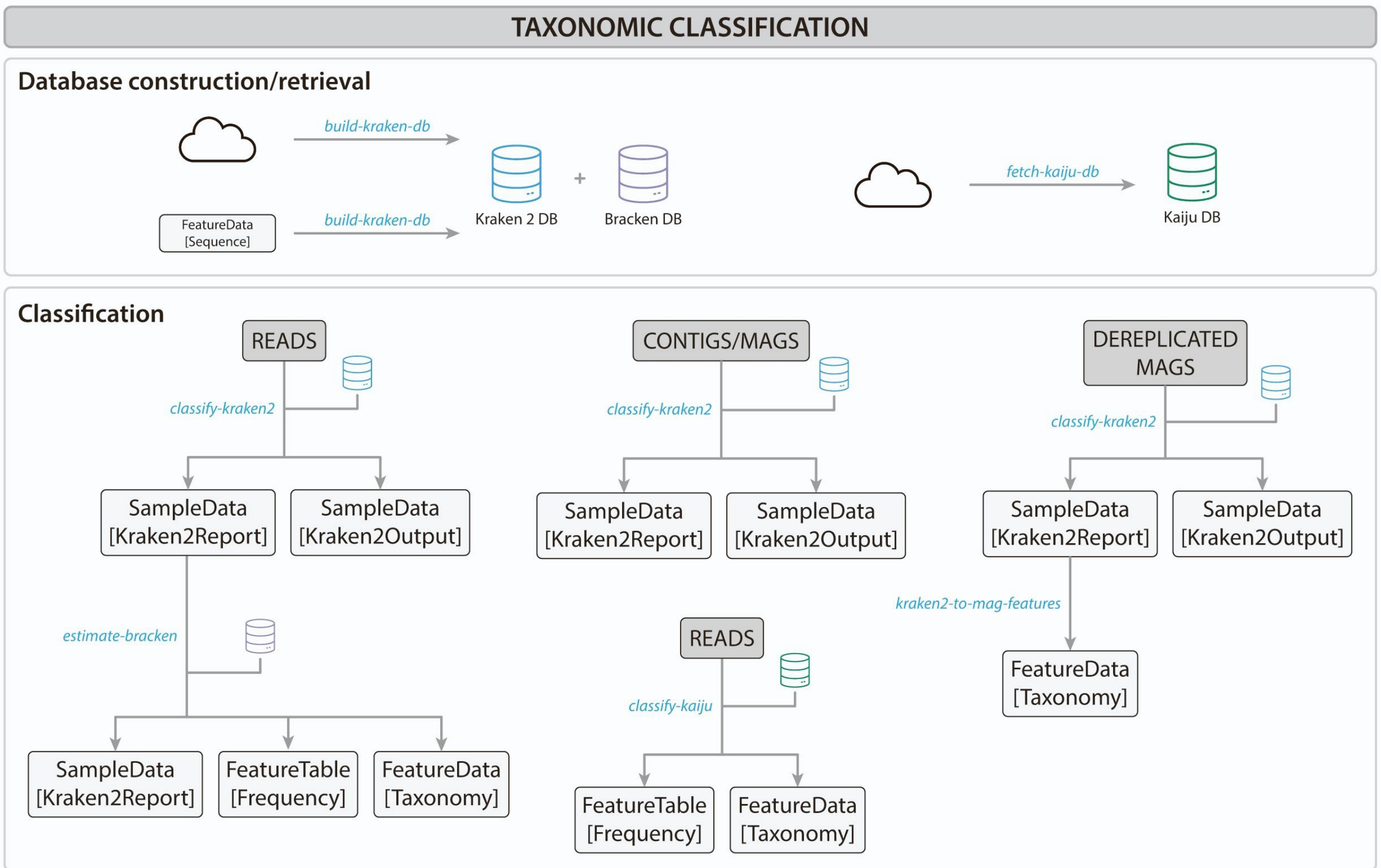

Figure S3: MOSHPIT taxonomic classification workflow, related to Figure 1A. Within the MOSHPIT plugin suite we provide two commonly used taxonomic classifiers: Kraken 2[8] together with its Bracken companion[9] used for read abundance correction and Kaiju for taxonomic classification[10]. We include actions which will allow users to easily fetch any of the pre-built databases for both of those classifiers (*build-kraken-db* and *fetch-kaiju-db* actions) and build custom Kraken 2 databases (*build-kraken-db*). We are planning on including an action to build custom Kaiju databases at a later point. Kraken 2 can be used to classify reads, contigs and MAGs (before and after dereplication) by using the *classify-kraken2* action. The reports obtained for the input reads can then be used as an input to the *estimate-bracken* action which will generate a feature table and a corresponding taxonomy. Furthermore, taxonomy features may be retrieved for the results generated from dereplicated MAGs by using the *kraken2-to-mag-features* - those can later be used to analyze the feature table obtained through MAG abundance estimation described in the previous section. The Kaiju classifier can be used to classify reads directly using the *classify-kaiju* action which will produce a feature table and a corresponding taxonomy, similar to the *classify-kraken2* action when reads are used as an input.

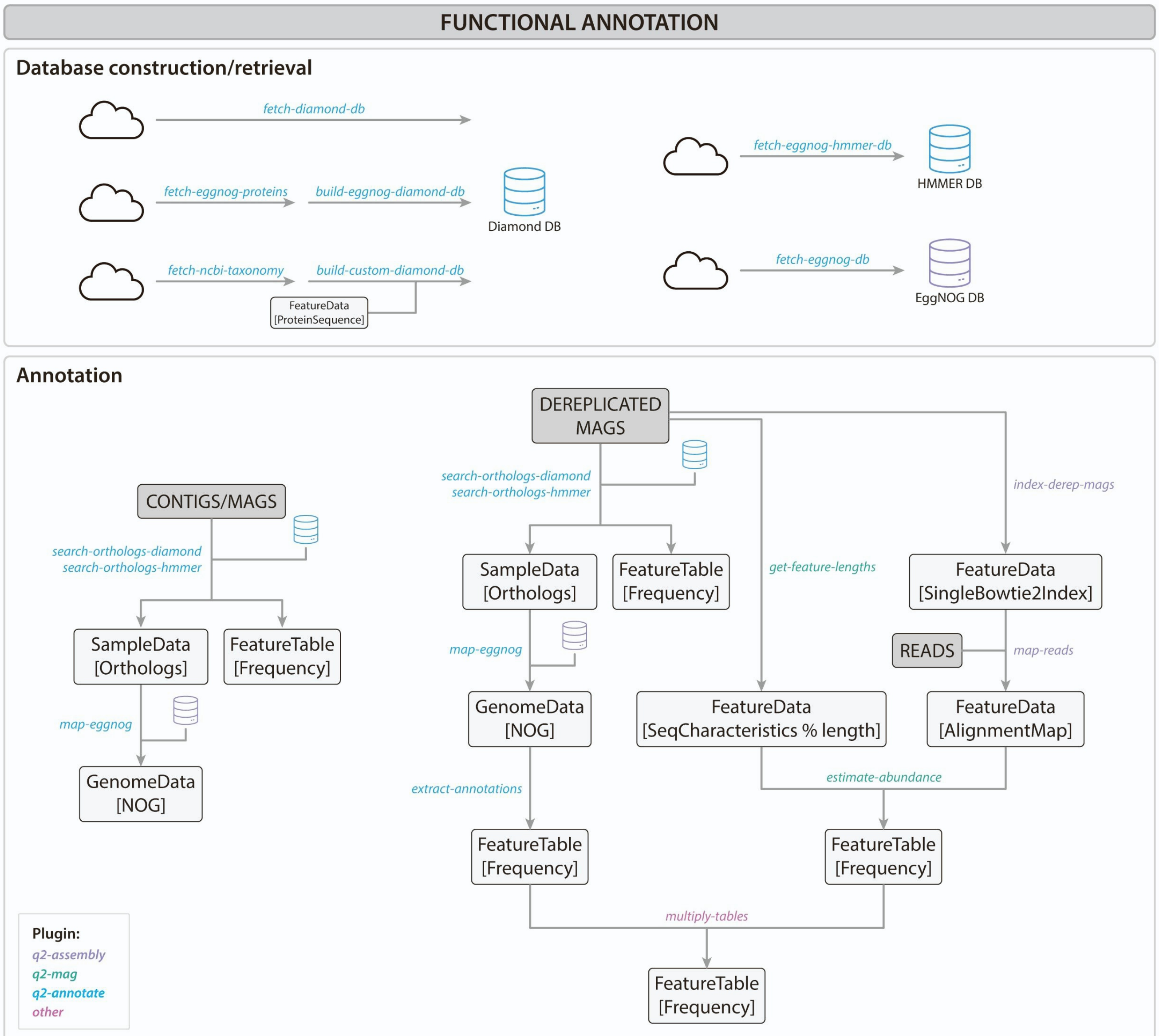

Figure S4. MOSHPIT functional annotation workflow, related to Figure 1A. The functional annotation workflow consists of various actions included in the q2-annotate plugin. The majority of these wrap EggNOG-mapper[11] to provide various options for functional annotation. We included several actions for retrieval/construction of databases for functional annotation, e.g., Diamond/HMMER databases for ortholog search and the EggNOG database used for ortholog annotation. Both contigs and MAGs (before and after dereplication) can be searched for protein orthologs using either *search-orthologs-diamond* or *search-orthologs-hmmer* actions. The resulting ortholog tables can then be annotated using the *map-eggnog* action and any specific annotation (e.g.: CAZymes or KEGG pathways) can be expanded into an independent feature table through the use of the *extract-annotations* action. This table can be passed as input into any other action accepting regular feature tables, e.g. to perform diversity analyses. Furthermore, when annotating the dereplicated MAGs (represented by the FeatureData[MAG] semantic type) this table can be combined with the feature table obtained from the MAG abundance estimation step using the *multiply-tables* action. This will generate a feature table with annotation counts normalized by the MAG abundances in each sample.

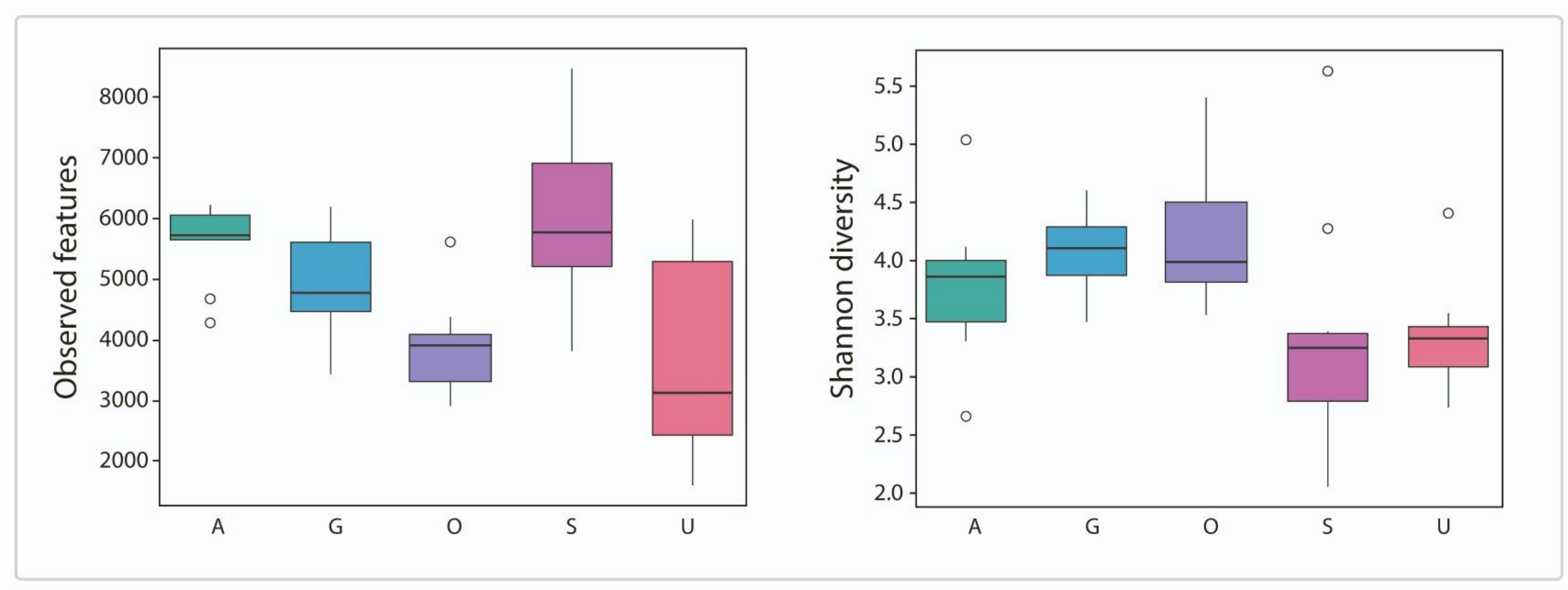

Figure S5: Alpha diversity analysis of each body site (A: airways, G: gastrointestinal tract, O: oral cavity, S: skin, U: urogenital tract), related to Figure 2.

Supplemental References

1. Liao, W.-W., Asri, M., Ebler, J., Doerr, D., Haukness, M., Hickey, G., Lu, S., Lucas, J.K., Monlong, J., Abel, H.J., et al. (2023). A draft human pangenome reference. Nature *617*, 312–324. https://doi.org/10.1038/s41586-023-05896-x.

2. Li, D., Liu, C.M., Luo, R., Sadakane, K., and Lam, T.W. (2015). MEGAHIT: An ultra-fast single-node solution for large and complex metagenomics assembly via succinct de Bruijn graph. Bioinformatics *31*, 1674–1676. https://doi.org/10.1093/bioinformatics/btv033.

3. Nurk, S., Meleshko, D., Korobeynikov, A., and Pevzner, P.A. (2017). MetaSPAdes: A new versatile metagenomic assembler. Genome Research *27*, 824–834. https://doi.org/10.1101/gr.213959.116.

4. Mikheenko, A., Saveliev, V., and Gurevich, A. (2016). MetaQUAST: Evaluation of metagenome assemblies. Bioinformatics *32*, 1088–1090. https://doi.org/10.1093/bioinformatics/btv697.

5. Kang, D.D., Li, F., Kirton, E., Thomas, A., Egan, R., An, H., and Wang, Z. (2019). MetaBAT 2: An adaptive binning algorithm for robust and efficient genome reconstruction from metagenome assemblies. PeerJ *2019*. https://doi.org/10.7717/peerj.7359.

6. Manni, M., Berkeley, M.R., Seppey, M., and Zdobnov, E.M. (2021). BUSCO: Assessing Genomic Data Quality and Beyond. Current Protocols *1*, e323. https://doi.org/10.1002/cpz1.323.

7. Pierce, N.T., Irber, L., Reiter, T., Brooks, P., and Brown, C.T. (2019). Large-scale sequence comparisons with *sourmash*. Preprint at F1000Research, https://doi.org/10.12688/f1000research.19675.1 https://doi.org/10.12688/f1000research.19675.1.

8. Wood, D.E., Lu, J., and Langmead, B. (2019). Improved metagenomic analysis with Kraken 2. Genome Biology *20*, 257. https://doi.org/10.1186/s13059-019-1891-0.

9. Lu, J., Breitwieser, F.P., Thielen, P., and Salzberg, S.L. (2017). Bracken: estimating species abundance in metagenomics data. PeerJ Comput. Sci. *3*, e104. https://doi.org/10.7717/peerj-cs.104.

10. Menzel, P., Ng, K.L., and Krogh, A. (2016). Fast and sensitive taxonomic classification for metagenomics with Kaiju. Nat Commun *7*, 11257. https://doi.org/10.1038/ncomms11257.

11. Huerta-Cepas, J., Forslund, K., Coelho, L.P., Szklarczyk, D., Jensen, L.J., von Mering, C., and Bork, P. (2017). Fast Genome-Wide Functional Annotation through Orthology Assignment by eggNOG-Mapper. Molecular Biology and Evolution *34*, 2115–2122. https://doi.org/10.1093/molbev/msx148.
